# Integrated multi-omics analysis identifies candidate genes and proteins associated with oleic acid accumulation in seedless oil palm

**DOI:** 10.3389/fpls.2026.1935397

**Published:** 2026-10-13

**Authors:** Zhoulian Li, Jerome Jeyakumar John Martin, Yongxiu Chen, Yuxin Liu, Cuilian Zhou, Qihong Li, Jiejun Huang, Xiaoyu Liu, Xinyu Li, Hongxing Cao, Shuanghong Cheng

**Affiliations:** 1 College of Tropical Crops, Yunnan Agricultural University, Pu’er, China; 2 Coconut Research Institute, Chinese Academy of Tropical Agricultural Sciences, Wenchang, China; 3 National Key Laboratory for Tropical Crop Breeding, Chinese Academy of Tropical Agricultural Sciences, Haikou, China

**Keywords:** candidate protein, fatty acid biosynthesis, lipid accumulation, multi-omics analysis, oleic acid, seedless oil palm

## Abstract

Oil palm (*Elaeis guineensis* Jacq.) is one of the world’s most important woody oil crops, with the mesocarp serving as the primary site of palm oil accumulation. Oleic acid (C18:1), an important monounsaturated fatty acid in palm oil, directly affects palm oil quality through changes in its abundance. Although seedless oil palm exhibits relatively high oleic acid levels, the molecular processes associated with its accumulation remain poorly understood. In this study, seedless oil palm mesocarp samples collected at 95 (FL1), 125 (FL2), and 185 (FL3) days after pollination were subjected to integrated metabolomic, proteomic, and transcriptomic analyses, followed by qRT-PCR analysis of selected candidate genes. Metabolomic and proteomic analyses included two biological replicates per developmental stage, whereas transcriptomic analysis was based on one biological sample per stage and qRT-PCR analysis included three biological replicates. The relative LC–MS/MS signal of oleic acid increased markedly during the late developmental stage (FL3). Multi-omics analyses revealed developmental changes in pathways associated with fatty acid biosynthesis, fatty acid metabolism, glycolysis/gluconeogenesis, pyruvate metabolism, carbon metabolism, and glycerolipid/glycerophospholipid metabolism. Proteomic KEGG analysis further showed significant enrichment of several carbon- and fatty-acid-related pathways after multiple-testing correction. Among the candidate genes and proteins identified, PDH E1α, DLAT, and ACC1 were mainly associated with precursor supply, including pyruvate-to-acetyl-CoA conversion and malonyl-CoA formation; KAS-related candidates, FabG-like, and FabZ were associated with chain elongation, reduction, and dehydration reactions in the fatty acid biosynthetic cycle; and LACS4, PLS1, and PLD1 were associated with fatty acid activation and downstream glycerolipid/glycerophospholipid metabolism. FAD2-like was further associated with the downstream desaturation of C18:1 to C18:2, linking oleic acid formation with subsequent C18-unsaturated fatty acid metabolism. Integrated analysis prioritized several candidate genes and proteins associated with oleic acid accumulation. qRT-PCR confirmed significant developmental differences for *SAD, FATB1*, and *FabZ*, whereas *PKp2*, *PFPα*, *PDH E1α, KAS12*, *ACC1*, and *EgCEK2-like* were supported mainly by pathway position and descriptive multi-omics patterns; *FabG-like, DLAT, LACS4, PLS1*, and *PLD1* were not examined by qRT-PCR. Among these candidates, SAD was associated with the C18:0-ACP-to-C18:1-ACP desaturation step, and its qRT-PCR expression showed a pronounced increase at FL3, consistent with the late-stage increase in the oleic-acid-associated LC–MS/MS signal. Collectively, this study identified metabolic pathways, candidate genes, and proteins associated with oleic acid accumulation in seedless oil palm mesocarp at the metabolite, transcript, and protein levels, providing candidate targets for subsequent functional validation.

## 1 Introduction

Oil palm (*Elaeis guineensis* Jacq.) is one of the world’s most productive tropical oil crops, contributing substantially to global vegetable oil production owing to its exceptionally high oil yield per unit area and considerable economic importance (Corley and Tinker, 2016; FAO, 2023; Afifi et al., 2025). The mesocarp is the primary site for palm oil accumulation, and its fatty acid composition strongly influences the nutritional quality, oxidative stability, and industrial applications of palm oil (Parveez et al., 2016; Martin et al., 2023; Afifi et al., 2025). Among the major fatty acids, oleic acid (C18:1) is particularly important because its high oxidative stability and favorable nutritional properties improve oil quality and extend shelf life (Saini and Keum, 2018; Xu et al., 2025a). Consequently, elucidating the molecular mechanisms that regulate oleic acid biosynthesis and accumulation during mesocarp development is essential for developing high-oleic oil palm germplasm and improving palm oil quality.

Oleic acid formation in plants involves coordinated processes of *de novo* fatty acid biosynthesis, stearoyl-ACP desaturation, acyl release, and glycerolipid assembly. *De novo* fatty acid biosynthesis occurs primarily in plastids and involves enzymes such as ACC, BC/BCCP, FabD, FabG, FabI, KAS, and FATB, which participate in acetyl-CoA carboxylation, malonyl-CoA formation, fatty acid chain elongation, reduction, dehydration, and acyl release (Li-Beisson et al., 2013; Bu et al., 2023; Afifi et al., 2025). Among these enzymes, stearoyl-ACP desaturase (SAD) catalyzes the conversion of stearoyl-ACP (C18:0-ACP) to oleoyl-ACP (C18:1-ACP), representing an important step in oleic acid biosynthesis (Li-Beisson et al., 2013; Miklaszewska et al., 2021; Bu et al., 2023; Gao et al., 2025). Acyl-ACP thioesterases such as *FATA* and *FATB* subsequently participate in fatty acid release, and *FATA*-associated regulation has also been implicated in oleic acid accumulation in oil palm (Luo et al., 2024). Following C18:1 formation, *FAD2*-mediated desaturation further links oleic acid with downstream C18-unsaturated fatty acid metabolism, and several studies have demonstrated the importance of *FAD2*-related genes in determining unsaturated fatty acid composition in oil palm mesocarp (Shi et al., 2021; Bahariah et al., 2023; Li et al., 2025).

Following fatty acid synthesis, enzymes such as LACS, GPAT, DGAT, PDAT, and PLD regulate acyl activation, triacylglycerol synthesis, glycerophospholipid metabolism, and lipid remodeling, thereby influencing the partitioning of oleoyl groups between storage lipids and membrane lipids (Regmi et al., 2020; Zhao et al., 2021; Sagun et al., 2023; Cai et al., 2025). Long-chain acyl-CoA synthetases have also been characterized in oil palm mesocarp and are involved in the activation of long-chain fatty acids prior to their entry into downstream lipid metabolic pathways (Wang et al., 2021). Previous studies have provided important insights into carbon metabolism, fatty acid biosynthesis, and oleic acid accumulation in oil palm mesocarp. Comparative transcriptomic and metabolomic analyses of oil palm and date palm showed that the oil-rich mesocarp of oil palm is characterized by enhanced expression of genes associated with plastidial carbon metabolism and fatty acid biosynthesis, including phosphofructokinase, pyruvate kinase, and pyruvate dehydrogenase (Bourgis et al., 2011). More recent studies have identified several genes associated with oleic acid accumulation, including *SAD*, *FabD*, *LACS6*, *BC*, *FabB*, and *FabI*, and have linked fatty acid composition with developmental and material-specific variation in oil palm (Martin et al., 2023; Xu et al., 2025a, 2025b). Comparative transcriptomic and metabolomic studies of seedless and tenera oil palm have further revealed differences in fatty acid synthesis and lipid-related metabolism between contrasting fruit types (Wei et al., 2024), while other multi-omics studies have highlighted coordinated changes in lipid metabolism across oil palm fruit development and related physiological processes (Hassan et al., 2024; Martin et al., 2024, 2025).

Transcriptomic and metabolomic analyses have also identified genes and metabolites associated with fatty acid metabolism and quality changes in oil palm fruit (Zhang et al., 2023). Studies in other oil-accumulating mesocarp tissues have likewise shown that changes in monounsaturated and polyunsaturated fatty acid accumulation are associated with coordinated developmental regulation of lipid metabolic pathways (Tsitsekian et al., 2025).

However, the relationships among metabolite accumulation, transcript profiles, and protein abundance during oleic acid accumulation in seedless oil palm mesocarp remain insufficiently characterized. Based on this, seedless oil palm mesocarp samples collected at 95, 125, and 185 days after pollination were subjected to metabolomic, transcriptomic, and proteomic analyses. The integrated analysis focused on metabolic processes associated with precursor supply, fatty acid biosynthesis and chain elongation, stearoyl-ACP desaturation, acyl release, and glycerolipid/glycerophospholipid metabolism. By comparing developmental changes across multiple molecular layers, this study aimed to characterize the pathways associated with oleic acid accumulation and to prioritize candidate genes, proteins, and metabolic pathway nodes for subsequent functional validation.

## 2 Materials and methods

### 2.1 Plant materials and sample collection

The seedless oil palm materials used in this study were cultivated at the Oil Palm Research Base of the Coconut Research Institute, Chinese Academy of Tropical Agricultural Sciences, located in Wenchang, Hainan Province, China (19°33′ N, 110°47′ E). The oil palms used for fruit sampling were established from seeds. Mesocarp tissues from seedless oil palm fruits were harvested at 95, 125, and 185 days after pollination and hereafter referred to as FL1, FL2, and FL3, respectively. These developmental stages were selected based on fruit developmental status, mesocarp formation, and the progression of oil and oleic acid accumulation. Earlier developmental stages were not included because the mesocarp was insufficiently developed for parallel multi-omics analyses, whereas later stages were avoided to minimize potential effects associated with over-maturation. At each developmental stage, fruits exhibiting comparable developmental characteristics were selected for analysis.

For each pooled sample, mesocarp tissues from five fruits collected from the same fruit bunch were combined. Two pooled-fruit biological replicates were prepared per developmental stage for metabolomic and proteomic analyses, one pooled-fruit biological sample per stage for transcriptomic analysis, and three pooled-fruit biological replicates per stage for qRT-PCR analysis. Immediately after harvest, mesocarp tissues were excised, homogenized under liquid nitrogen, divided into aliquots for metabolomic, proteomic, transcriptomic, and qRT-PCR analyses, rapidly frozen in liquid nitrogen, and stored at −80 °C until further analysis.

### 2.2 Metabolomic analysis and data analysis

Untargeted metabolomic profiling was performed using liquid chromatography–tandem mass spectrometry (LC–MS/MS) with two biological replicates per developmental stage. Samples were thawed on ice, and metabolites were extracted with pre-cooled methanol/acetonitrile/water (2:2:1, v/v/v). Following low-temperature sonication and centrifugation, the supernatants were collected, vacuum-dried, and reconstituted in acetonitrile/water (1:1, v/v) prior to LC–MS/MS analysis. Quality control (QC) samples were prepared by pooling equal aliquots from all biological samples and were analyzed throughout the analytical sequence. Chromatographic separation was performed using hydrophilic interaction liquid chromatography (HILIC), with mobile phase A consisting of 25 mM ammonium acetate and 25 mM ammonia in water and mobile phase B consisting of acetonitrile.

Raw mass spectrometry data were converted using ProteoWizard and processed using XCMS for peak detection, retention-time correction, peak alignment, and peak-area extraction. Metabolite features with more than 50% missing values within any sample group were excluded, and the remaining missing values were imputed using the k-nearest neighbor (KNN) algorithm, followed by total peak-area normalization. The resulting metabolomic data were expressed as normalized relative LC–MS/MS signals. PLS-DA and OPLS-DA were performed using the ropls package in R, and OPLS-DA models were evaluated by cross-validation and permutation testing. Variable importance in projection (VIP) values were obtained from the OPLS-DA models. For pairwise comparisons, P values were obtained using univariate t-tests and subsequently adjusted for multiple testing using the Benjamini–Hochberg procedure. Differentially accumulated metabolites (DAMs) were screened using VIP ≥ 1, |log_2_FC| ≥ 1, and FDR < 0.05. The union of FDR-screened DAMs from the three pairwise developmental comparisons was used for Venn analysis, K-means clustering, functional annotation, and KEGG pathway analysis. KEGG pathway enrichment was evaluated using a hypergeometric test followed by Benjamini–Hochberg correction.

### 2.3 Transcriptome sequencing and data analysis

Total RNA was extracted from frozen mesocarp tissues using the Plant Total RNA Isolation Kit for polysaccharide- and polyphenol-rich samples (TIANGEN Biotech, Beijing, China; Cat. No. DP441) according to the manufacturer’s instructions. RNA quality and integrity were assessed using 1% agarose gel electrophoresis, a NanoDrop 2000 spectrophotometer (Thermo Fisher Scientific, USA), and an Agilent 2100 Bioanalyzer (Agilent Technologies, USA). mRNA libraries were prepared using the Hieff NGS^®^ Ultima Dual-mode mRNA Library Prep Kit for Illumina^®^ (Yeasen Biotechnology, Shanghai, China; Cat. No. 12309ES) and sequenced on the Illumina NovaSeq X Plus platform (Illumina, San Diego, CA, USA) using 150-bp paired-end sequencing.

Raw reads were processed using fastp (Chen et al., 2018) to remove adapter sequences and low-quality reads. Ribosomal RNA sequences were removed using Bowtie2 (Langmead and Salzberg, 2012), and the remaining clean reads were aligned to the Elaeis guineensis reference genome (NCBI RefSeq assembly GCF_000442705.1) using HISAT2 (Kim et al., 2019). Transcript assembly and expression quantification were performed using StringTie (Pertea et al., 2015) and RSEM (Li and Dewey, 2011), and transcript abundance was expressed as fragments per kilobase of transcript per million mapped reads (FPKM).

One biological sample was analyzed for each developmental stage. Therefore, statistical differential-expression analysis was not performed. For descriptive developmental analysis, genes with a maximum FPKM ≥ 1 and a maximum-minus-minimum difference ≥ 1 after log_2_(FPKM + 1) transformation were retained. A total of 7,490 genes met these criteria and were used for heatmap visualization, K-means clustering, functional annotation, and pathway-level analysis.

### 2.4 Proteomic analysis and data analysis

Proteomic analysis was performed using two biological replicates per developmental stage. Total proteins were extracted from mesocarp tissues and processed using the iST sample preparation kit (PreOmics, Germany). After denaturation, reduction, and alkylation, proteins were digested with trypsin, followed by peptide desalting and vacuum drying. The dried peptides were reconstituted in 0.1% (v/v) formic acid prior to LC–MS/MS analysis. Peptides were separated using an UltiMate 3000 nano-LC system (Thermo Fisher Scientific, USA) coupled to a timsTOF Pro2 mass spectrometer (Bruker Daltonics, Germany). Chromatographic separation was performed on an Aurora C18 column (15 cm × 75 μm i.d., 1.7 μm particle size, 120 Å pore size; IonOpticks) at a flow rate of 400 nL min^−1^. Mass spectrometric data were acquired in diaPASEF mode (Meier et al., 2020) over an m/z range of 349–1229 using an isolation window width of 40 Da. Protein identification and label-free quantification were performed using Spectronaut 14 (Biognosys AG, Switzerland) with the DirectDIA workflow (Bruderer et al., 2017; Gotti et al., 2021). Indexed retention time (iRT) calibration was applied for retention-time normalization during DIA data processing (Escher et al., 2012). Protein identifications were controlled at a false discovery rate (FDR) of 1% at both the precursor and protein levels. Normalized label-free protein abundance values derived from peptide/protein MS signal intensities were used for downstream statistical analyses and represented relative protein abundance rather than absolute protein concentration. Within-stage biological-replicate concordance was assessed for proteins quantified in both replicates. Pearson correlation coefficients were calculated using log_2_(normalized protein abundance + 1) values. Coefficients of variation (CVs) were calculated from the normalized protein abundance values as the standard deviation divided by the mean and expressed as percentages. For pairwise comparisons between developmental stages, P values were calculated using a two-sided Student’s t-test based on two biological replicates per stage and were subsequently adjusted for multiple testing using the Benjamini–Hochberg procedure. Differentially abundant proteins (DAPs) were defined using |log_2_FC| ≥ 1 and FDR < 0.05. The resulting FDR-screened DAPs were used for subsequent clustering and KEGG pathway enrichment analyses.

### 2.5 Multi-omics integrated analysis

Based on KEGG annotation, functional annotation, biochemical pathway position, and developmental expression/abundance patterns, metabolomic, proteomic, and transcriptomic data were integrated to identify candidate genes, proteins, and metabolic pathway nodes associated with oleic acid accumulation during seedless oil palm mesocarp development. FDR-screened differentially accumulated metabolites (DAMs), FDR-screened differentially abundant proteins (DAPs), and genes showing developmental FPKM variation were mapped to pathways related to precursor supply, fatty acid biosynthesis and chain elongation, stearoyl-ACP desaturation, acyl release and activation, and glycerolipid/glycerophospholipid metabolism. Candidate genes and proteins were prioritized according to their pathway functions, developmental patterns, and consistency across omics layers, with qRT-PCR results used as additional supporting evidence where available.

### 2.6 Statistical analysis

Statistical analyses were performed using IBM SPSS Statistics (version 25; IBM Corp., Armonk, NY, USA), GraphPad Prism (version 9.5; GraphPad Software, San Diego, CA, USA), and the Metware Cloud platform. Data are presented as the mean ± standard deviation (SD) of biological replicates. Metabolomic and proteomic analyses were based on two biological replicates per developmental stage. Multiple-testing correction was performed using the Benjamini–Hochberg procedure, and the corresponding FDR-based screening criteria are described in Sections 2.2 and 2.4.

Because only two biological replicates were available per developmental stage, the selected fatty-acid-related LC–MS/MS signals shown in **Figure 1** were summarized descriptively using individual biological-replicate values and mean ± SD. For RNA-seq, one biological sample was analyzed per developmental stage, and transcriptomic data were used for descriptive developmental FPKM analysis without statistical differential-expression testing. For qRT-PCR, three biological replicates were analyzed per developmental stage. Relative transcript levels were calculated using the 2^−ΔΔCt method (Livak and Schmittgen, 2001) with Actin as the reference gene and statistical comparisons were performed on ΔCt values using one-way ANOVA followed by Tukey’s multiple-comparison test. A value of P < 0.05 was considered statistically significant. Heatmaps, Venn diagrams, K-means clustering plots, and other omics visualizations were generated using the Metware Cloud platform and GraphPad Prism as appropriate.

**Figure 1:**
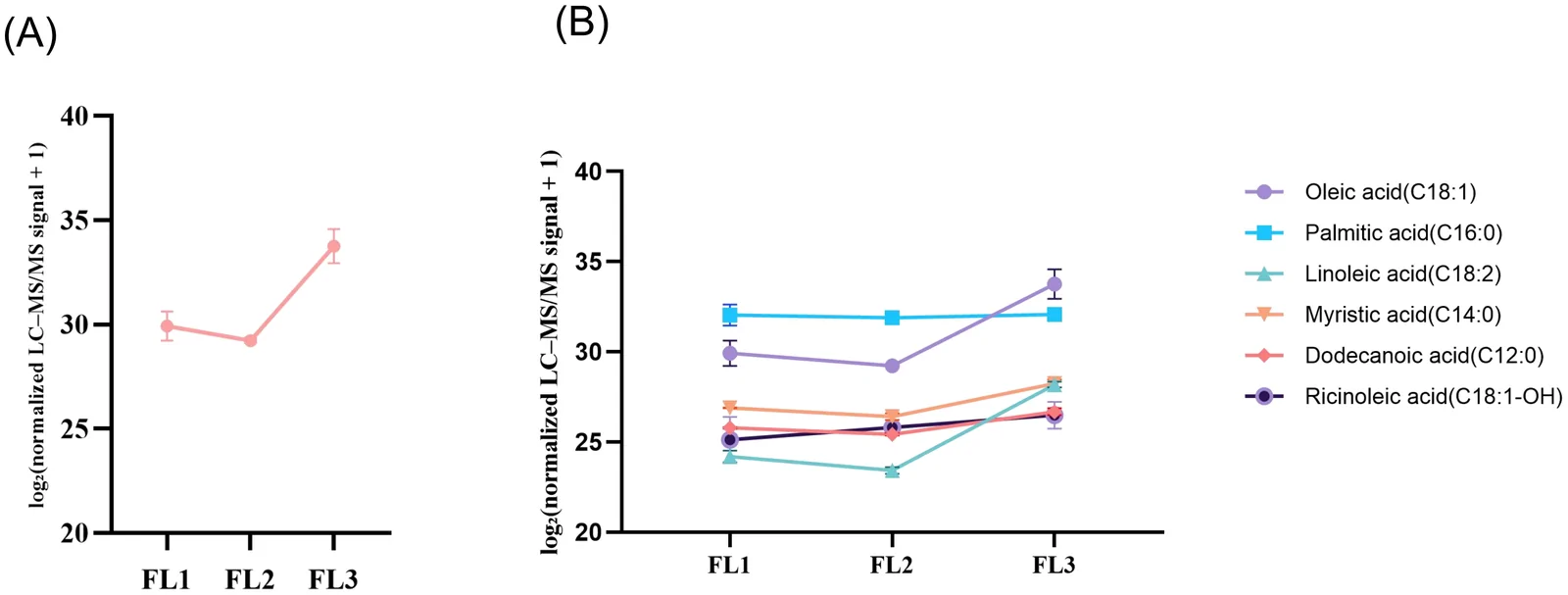
Developmental patterns of oleic acid and selected fatty-acid-related LC–MS/MS signals during seedless oil palm mesocarp development. **(A)** Normalized LC–MS/MS signal of oleic acid across FL1, FL2, and FL3, shown as log_2_(normalized signal + 1). **(B)** Developmental patterns of oleic acid (C18:1), palmitic acid (C16:0), linoleic acid (C18:2), myristic acid (C14:0), dodecanoic acid (C12:0), and ricinoleic acid (C18:1-OH). These six annotated fatty-acid-related features were selected to illustrate different carbon-chain lengths, degrees of unsaturation, and contrasting developmental patterns, with oleic acid as the focal fatty acid of this study; they do not represent the complete fatty-acid-related feature set detected in the untargeted metabolomic analysis. Data are presented as mean ± SD of two biological replicates per developmental stage. The LC–MS/MS signals are interpreted descriptively because of the limited biological replication. A complete list of annotated fatty-acid-related features is provided in **Supplementary Table S1**.

## 3 Results and analysis

### 3.1 Developmental phenotypes and fatty-acid profiles of seedless oil palm mesocarp

Seedless oil palm fruits showed distinct developmental changes across FL1, FL2, and FL3, with the mesocarp gradually changing from pale yellow at FL1 to deeper yellow-orange at the later developmental stages (**Figure 2**).

**Figure 2:**
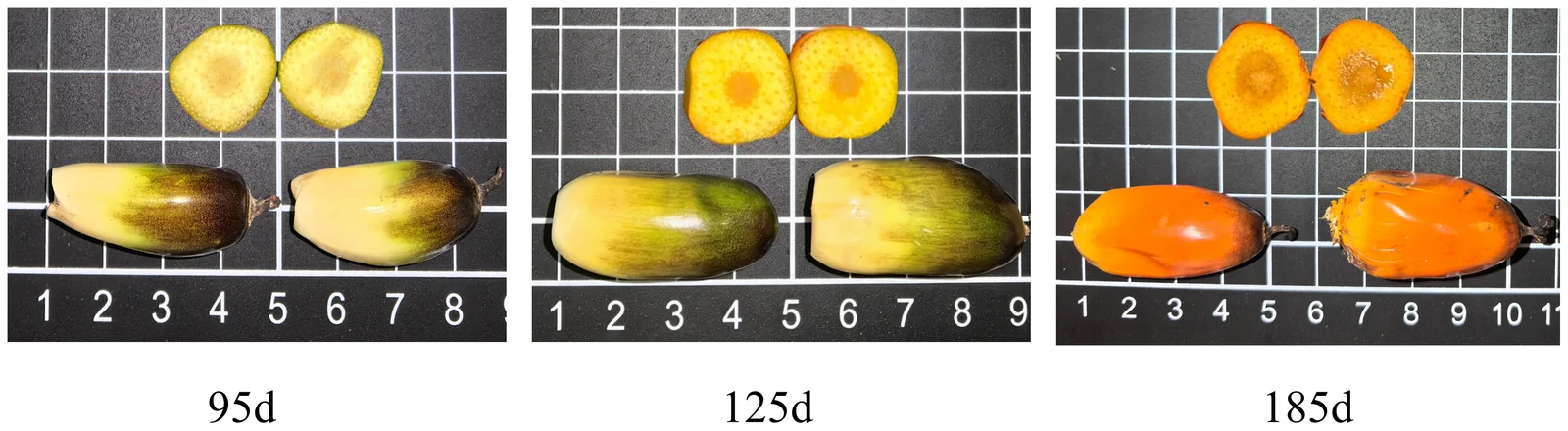
Representative fruit and mesocarp phenotypes of seedless oil palm at different developmental stages. Representative whole fruits and transverse mesocarp sections at FL1 (95 days after pollination), FL2 (125 days after pollination), and FL3 (185 days after pollination), showing developmental changes in fruit and mesocarp appearance during sampling.

Untargeted metabolomic analysis revealed distinct developmental patterns of fatty-acid-related LC–MS/MS signals (**Figure 1**). The normalized oleic-acid-associated signal decreased from FL1 to FL2 and subsequently showed a pronounced increase at FL3, reaching its highest level at the late developmental stage (**Figure 1A**). Other representative fatty acids exhibited different developmental patterns (**Figure 1B**). Linoleic acid (C18:2) decreased from FL1 to FL2 and subsequently increased at FL3, whereas palmitic acid (C16:0), myristic acid (C14:0), dodecanoic acid (C12:0), and ricinoleic acid (C18:1-OH) showed distinct stage-dependent changes. Overall, the late developmental stage was characterized by a pronounced increase in the oleic-acid-associated LC–MS/MS signal, providing a basis for subsequent analysis of metabolic pathways and genes and proteins associated with oleic acid accumulation.

### 3.2 Overall distribution of multi-omics data during seedless oil palm mesocarp development

Metabolomic PCA showed stage-associated distribution patterns among FL1, FL2, and FL3, with PC1 and PC2 explaining 59.09% and 19.91% of the total variance, respectively, and together accounting for 79.00% of the variance (**Figure 3A**). A similar stage-associated distribution pattern was observed in the proteomic PCA, in which PC1 and PC2 explained 53.68% and 25.35% of the total variance, respectively, with a cumulative contribution of 79.03% (**Figure 3B**). Hierarchical heatmap analysis of the 7,490 retained genes showed developmental variation in FPKM patterns across FL1, FL2, and FL3, including groups with relatively higher FPKM values at FL1, FL2, or FL3 (**Figure 3C**).

**Figure 3:**
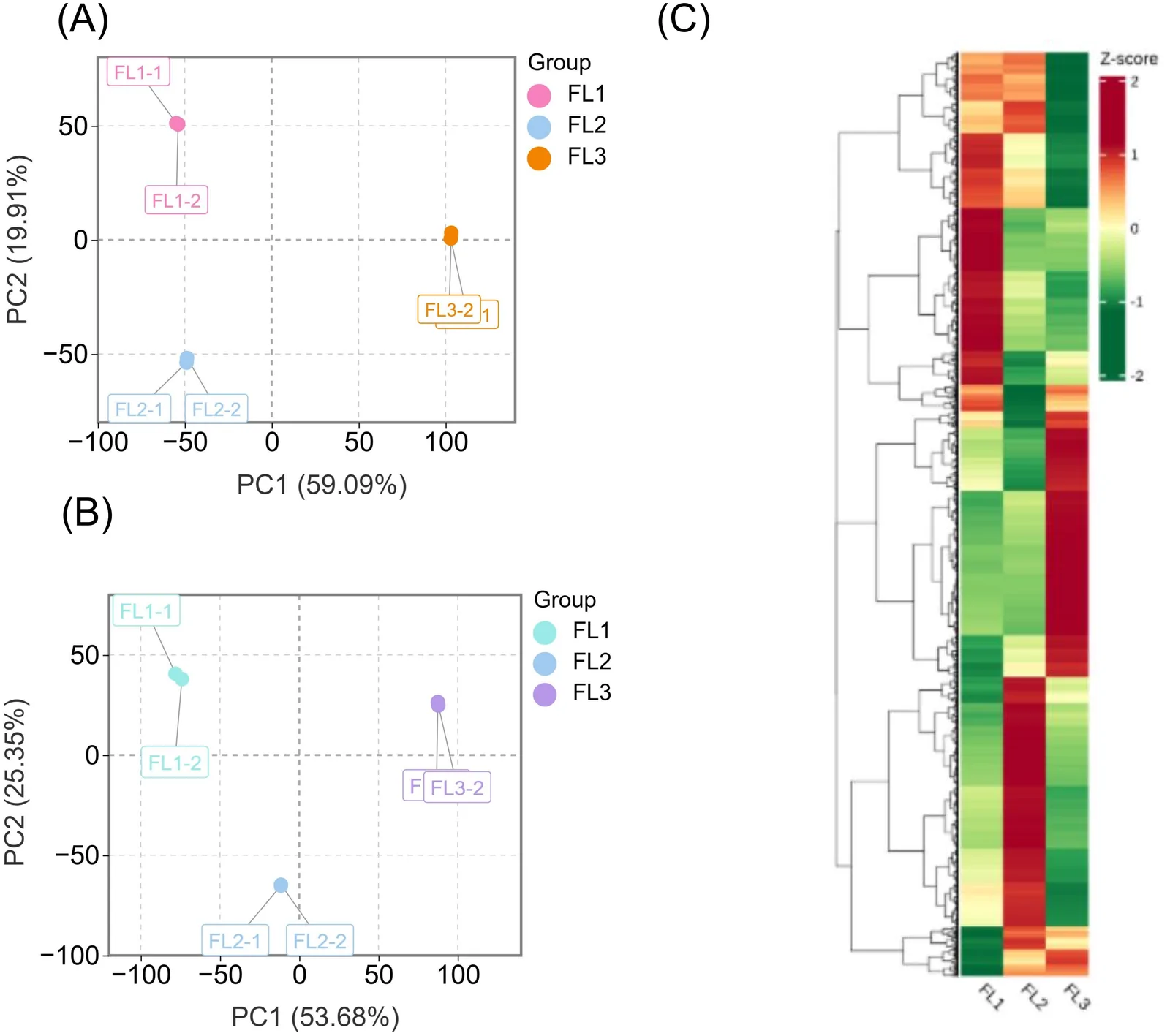
Overall distribution of multi-omics profiles during seedless oil palm mesocarp development. **(A)** Principal component analysis (PCA) of the metabolomic dataset. **(B)** PCA of the proteomic dataset. Each point in panels A and B represents one biological replicate, with two biological replicates per developmental stage. **(C)** Hierarchical heatmap of 7,490 genes retained for transcriptomic analysis. FPKM values were log_2_(FPKM + 1)-transformed and standardized by gene using Z-score transformation. Columns represent FL1, FL2, and FL3 in developmental order. RNA-seq was based on one biological sample per developmental stage.

### 3.3 Screening of differentially accumulated metabolites during seedless oil palm mesocarp development

Using VIP ≥ 1, |log_2_FC| ≥ 1, and FDR < 0.05 as the screening criteria, differentially accumulated metabolites were identified across pairwise comparisons of seedless oil palm at different developmental stages. A total of 264 differential metabolites, including 122 upregulated and 142 downregulated metabolites, were identified in FL1 vs FL2. In FL1 vs FL3, 743 differential metabolites were detected, of which 351 were upregulated and 392 were downregulated. In FL2 vs FL3, 714 differential metabolites were identified, including 365 upregulated and 349 downregulated metabolites (**Figures 4A–C**). The numbers of differential metabolites identified in FL1 vs FL3 and FL2 vs FL3 were markedly higher than that identified in FL1 vs FL2.

**Figure 4:**
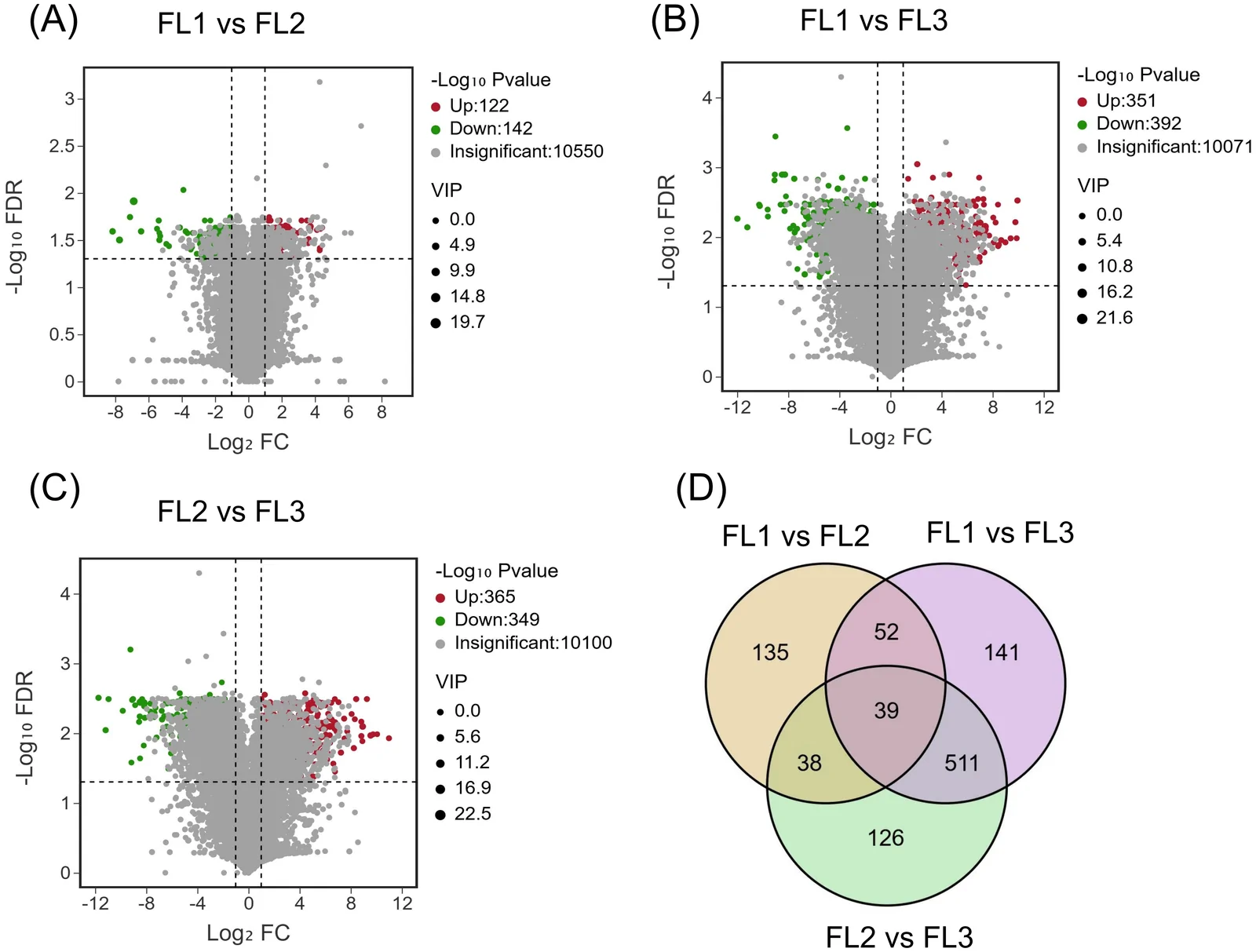
Analysis of differentially accumulated metabolites during seedless oil palm mesocarp development. **(A–C)** Volcano plots of differential metabolites for FL1 vs FL2, FL1 vs FL3, and FL2 vs FL3 **(D)** Venn diagram of differential metabolites among the three comparisons. Red and green points indicate upregulated and downregulated metabolites, respectively, and gray points indicate metabolites that did not meet the screening criteria. Values in the Venn diagram indicate the numbers of unique or shared differential metabolites.

Venn analysis showed that 39 differential metabolites were shared among all three comparisons, while 511 metabolites were shared exclusively between FL1 vs FL3 and FL2 vs FL3 (**Figure 4D**). In total, 1,042 differential metabolites were obtained from the union of the three pairwise comparisons and were subsequently used for K-means clustering and further functional analysis.

### 3.4 K-means clustering analysis of differential metabolites during seedless oil palm mesocarp development

K-means clustering divided the 1,042 differential metabolites into six subclasses with distinct developmental accumulation patterns across FL1, FL2, and FL3 (**Figure 5**). Subclasses 1–6 contained 151, 295, 147, 41, 49, and 359 metabolites, respectively. Subclasses 1 and 2 showed overall decreasing patterns toward FL3, whereas Subclass 3 showed relatively higher abundance at FL2. Subclass 4 exhibited a progressive increase across development, Subclass 5 decreased at FL2 followed by recovery at FL3, and Subclass 6 showed a marked increase at FL3.

**Figure 5:**
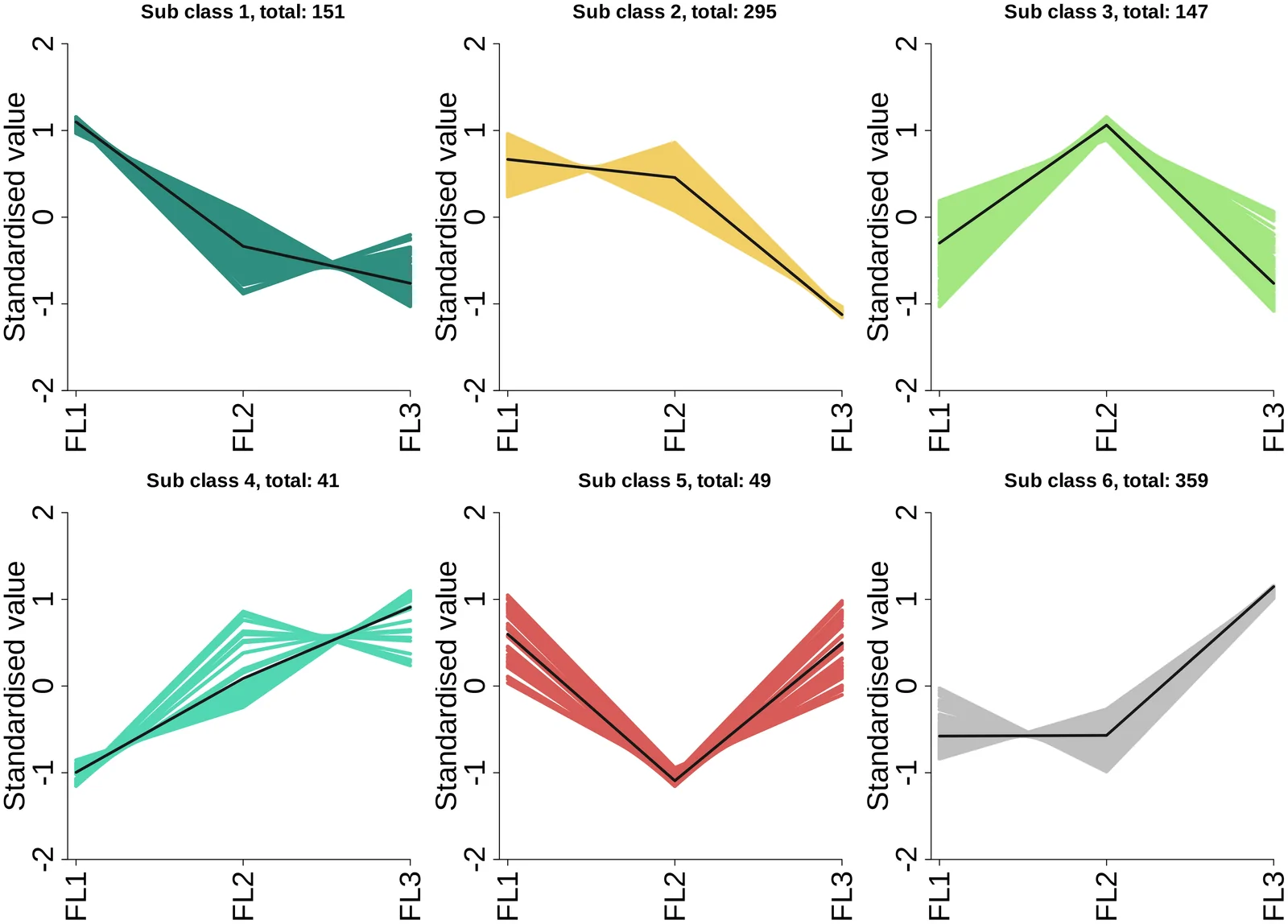
K-means clustering analysis of differentially accumulated metabolites during seedless oil palm mesocarp development. K-means clustering divided 1,042 differential metabolites into six subclasses with distinct developmental accumulation patterns across FL1, FL2, and FL3. The number in each panel indicates the number of metabolites assigned to the corresponding subclass, and the vertical axis represents standardized metabolite abundance. Oleic acid was not included in the K-means clustering because it did not meet the DAM screening criteria in any of the three pairwise comparisons.

Oleic acid was not assigned to a K-means subclass because it did not meet the DAM screening criteria used for clustering, primarily due to adjusted FDR values > 0.05. Its developmental LC–MS/MS signal pattern was therefore analyzed separately (**Figure 1**).

### 3.5 Developmental patterns of fatty-acid- and lipid-related metabolites

Among the 74 lipid- and lipid-like differentially accumulated metabolites (DAMs), 37 were assigned to Subclass 6 and 22 to Subclass 2 (**Figure 6A**). Among the 31 metabolites classified as Fatty Acyls, 17 were distributed in Subclass 6 and 8 in Subclass 2, with the remaining metabolites distributed among Subclasses 1, 3, 4, and 5.

**Figure 6:**
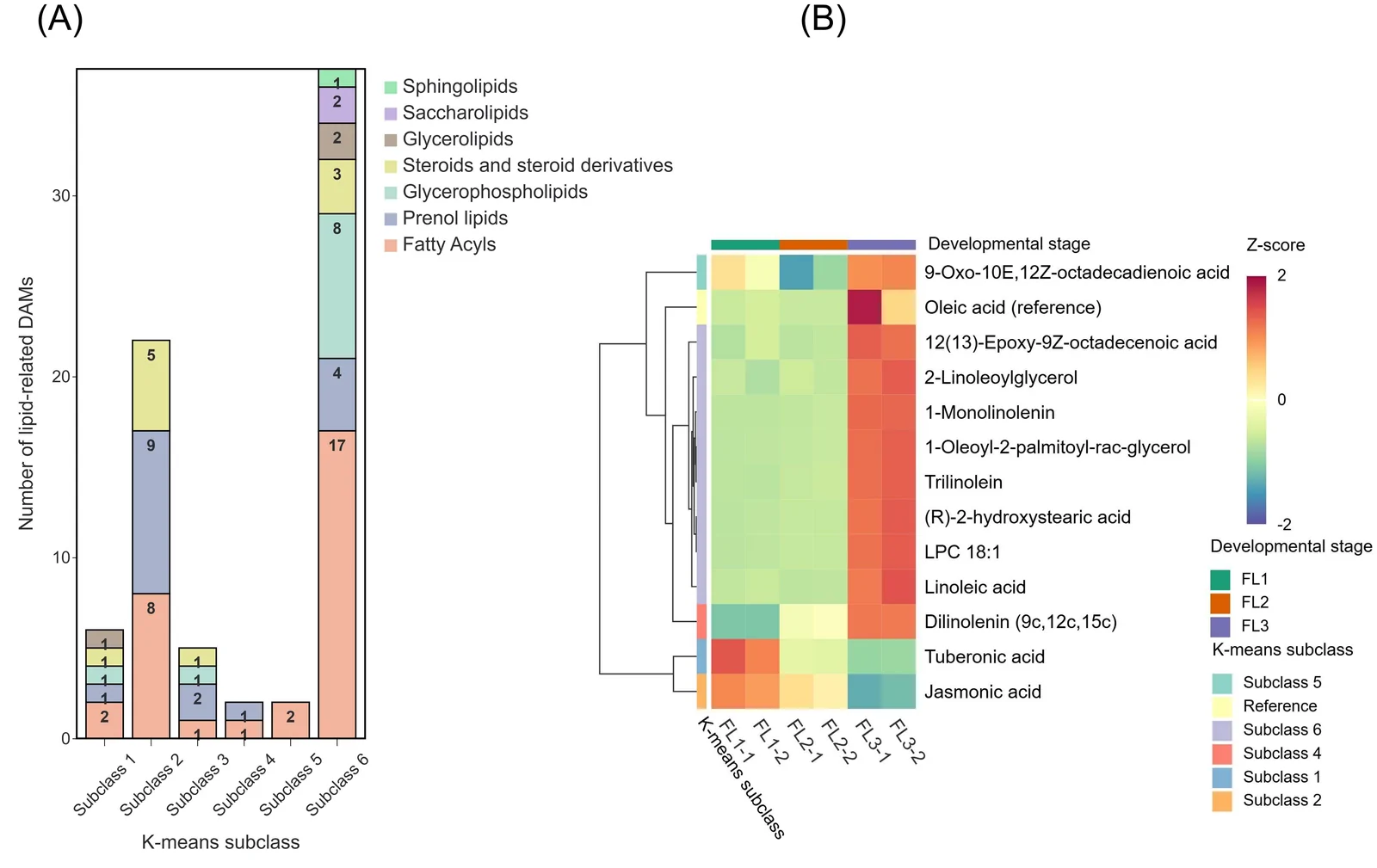
Developmental patterns of fatty-acid- and lipid-related metabolites in seedless oil palm mesocarp. **(A)** Distribution of lipid-related DAMs among the six K-means subclasses. Stacked bars show the numbers of metabolites assigned to different lipid classes in each subclass. **(B)** Heatmap showing the developmental abundance patterns of representative fatty-acid- and lipid-related metabolites across FL1, FL2, and FL3. The annotation bar indicates the K-means subclass assigned to each metabolite. Oleic acid is shown as a reference and was not included in the K-means clustering.

Representative metabolites showed distinct developmental accumulation patterns (**Figure 6B**). Linoleic acid, 12(13)-epoxy-9Z-octadecenoic acid, (R)-2-hydroxystearic acid, 1-monolinolenin, 2-linoleoylglycerol, LPC 18:1, and trilinolein showed relatively higher abundance at FL3. In contrast, tuberonic acid and jasmonic acid decreased during development, whereas dilinolenin showed a progressive increase and 9-oxo-10E,12Z-octadecadienoic acid decreased at FL2 followed by an increase at FL3.

### 3.6 Differential protein analysis during seedless oil palm mesocarp development

A total of 10,134 proteins were quantified in the proteomic dataset. Pearson correlation coefficients between biological replicates were 0.978, 0.982, and 0.980 for FL1, FL2, and FL3, based on 6,599, 8,170, and 7,960 proteins, respectively (**Supplementary Figure S1A–C**). The corresponding median CV values were 8.52%, 7.29%, and 6.85% (**Supplementary Figure S1D**), indicating high concordance between biological replicates. Using |log_2_FC| ≥ 1 and FDR < 0.05 as the screening criteria, differentially abundant proteins (DAPs) were identified among the three pairwise developmental comparisons. A total of 947 DAPs were identified in FL1 vs FL2, including 597 proteins with increased abundance and 350 with decreased abundance; 2,969 DAPs were identified in FL1 vs FL3, including 1,713 increased and 1,256 decreased proteins; and 1,952 DAPs were identified in FL2 vs FL3, including 1,025 increased and 927 decreased proteins (**Figures 7A–C**). Among the three comparisons, FL1 vs FL3 contained the largest number of DAPs.

**Figure 7:**
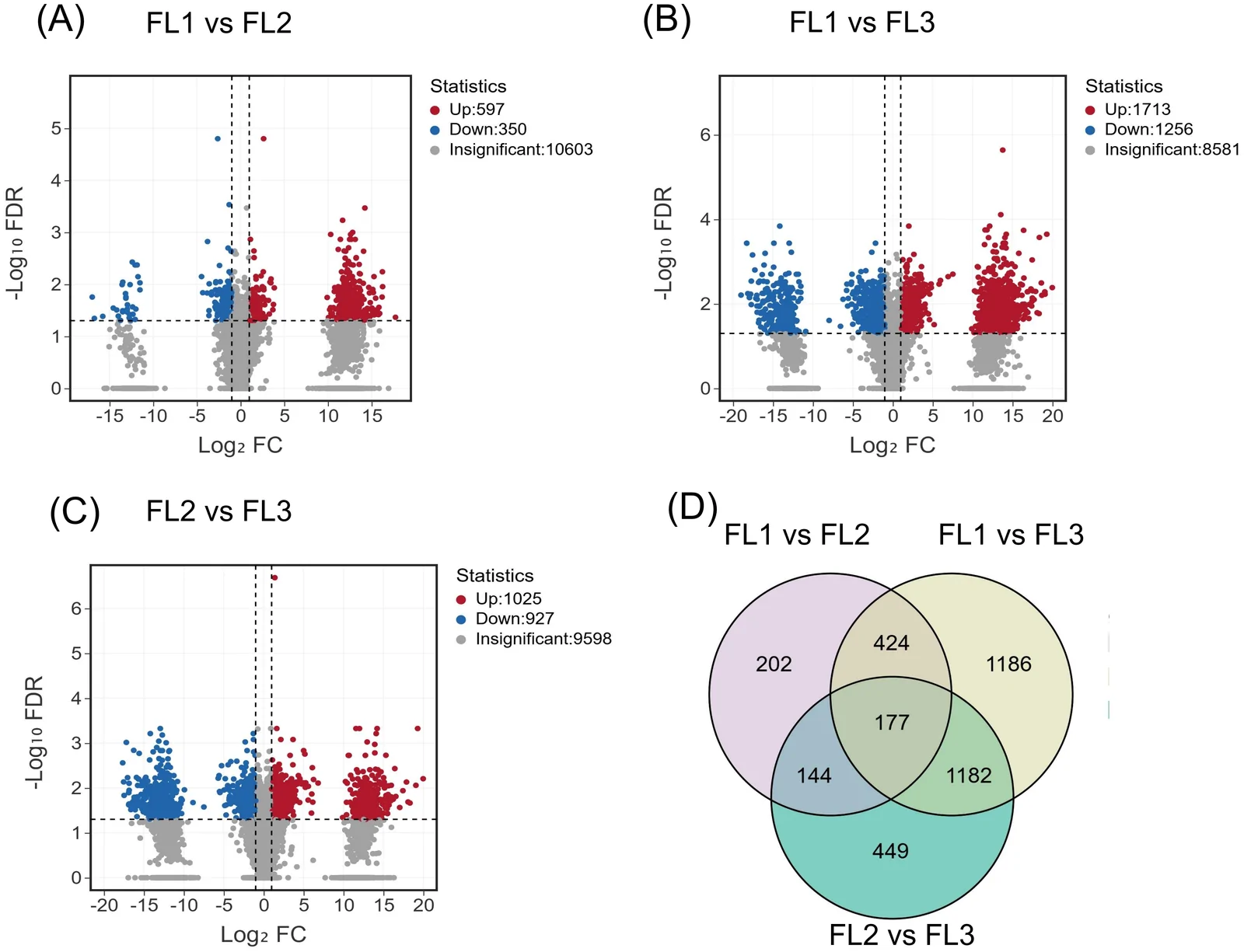
Differential protein abundance during seedless oil palm mesocarp development. Volcano plots of differentially abundant proteins in **(A)** FL1 vs FL2, **(B)** FL1 vs FL3, and **(C)** FL2 vs FL3. DAPs were identified using |log_2_FC| ≥ 1 and FDR < 0.05. Red and blue points indicate proteins with increased and decreased abundance, respectively, and gray points indicate non-significant proteins. **(D)** Venn diagram showing shared and comparison-specific DAPs among the three developmental comparisons.

Venn analysis showed that 177 DAPs were shared among all three comparisons. In addition, 424 DAPs were shared between FL1 vs FL2 and FL1 vs FL3, 144 between FL1 vs FL2 and FL2 vs FL3, and 1,182 between FL1 vs FL3 and FL2 vs FL3 (**Figure 7D**).

### 3.7 K-means clustering analysis of differentially abundant proteins during seedless oil palm mesocarp development

K-means clustering divided the 3,764 differentially abundant proteins into six subclasses with distinct abundance patterns across FL1, FL2, and FL3 (**Figure 8**). Subclasses 1–6 contained 143, 778, 1,166, 679, 269, and 729 proteins, respectively.

**Figure 8:**
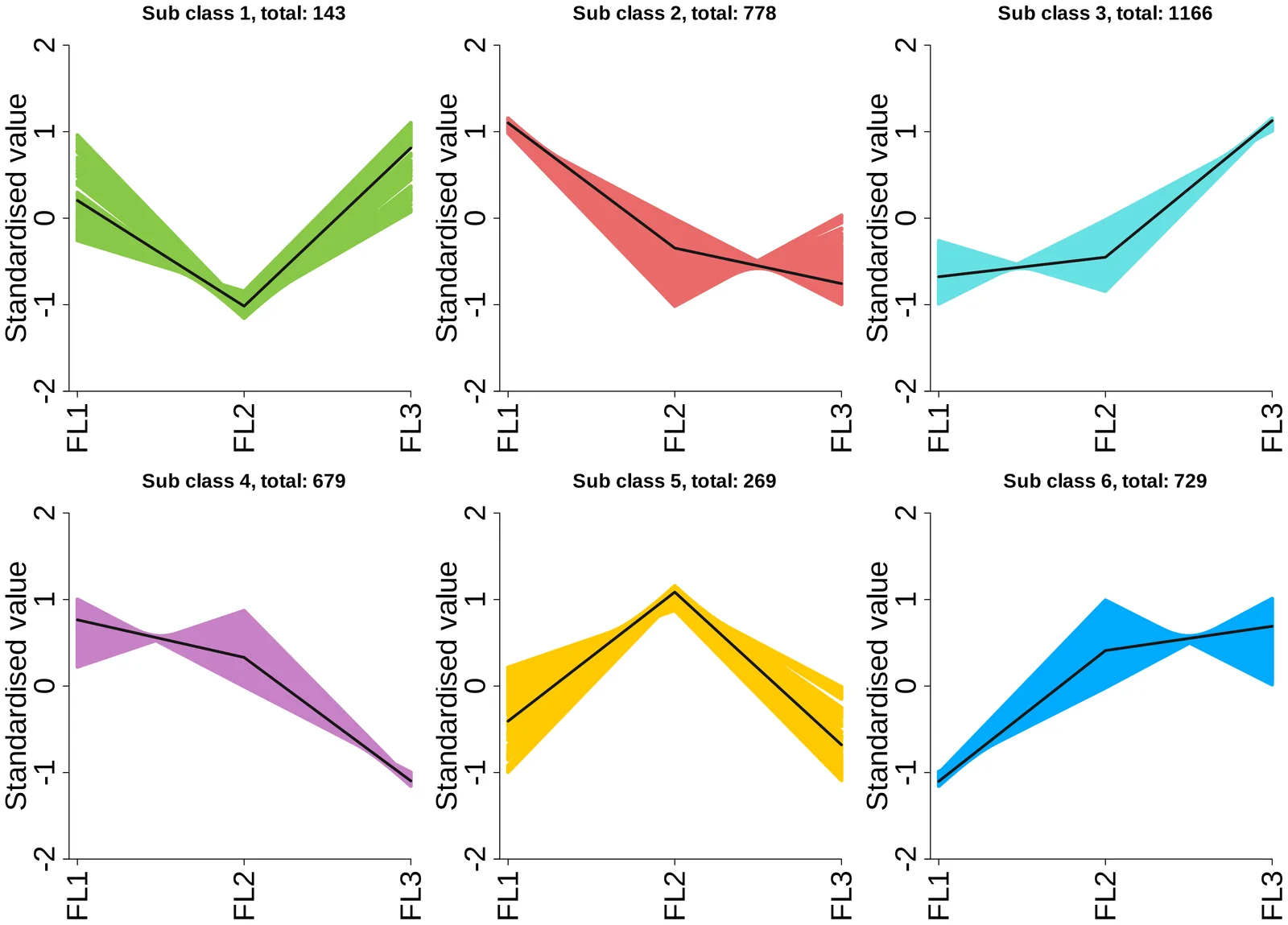
K-means clustering analysis of differentially abundant proteins during seedless oil palm mesocarp development. Six subclasses were identified based on the abundance patterns of 3,764 differentially abundant proteins across FL1, FL2, and FL3. The number in each panel indicates the number of proteins in each subclass, and the vertical axis represents standardized protein abundance. Proteomic analysis included two biological replicates per developmental stage.

Subclass 1 showed decreased abundance at FL2 followed by recovery at FL3, whereas Subclass 2 exhibited an overall decreasing pattern during development. Subclass 3 showed a marked increase at FL3, while Subclass 4 maintained relatively high abundance at FL1 and FL2 before decreasing at FL3. Subclass 5 showed relatively higher abundance at FL2, whereas Subclass 6 increased from FL1 to FL2 and remained relatively high at FL3.

### 3.8 Developmental patterns of fatty-acid- and lipid-metabolism-related proteins in seedless oil palm

Fatty-acid- and lipid-metabolism-related DAPs were distributed across all six K-means subclasses and were mainly associated with fatty acid biosynthesis, unsaturated fatty acid biosynthesis, fatty acid metabolism/degradation, glycerolipid metabolism, glycerophospholipid metabolism, and linoleic/α-linolenic acid metabolism (**Figure 9A**). Most of these proteins were assigned to Subclasses 3 and 6, with smaller numbers distributed among the remaining subclasses.

**Figure 9:**
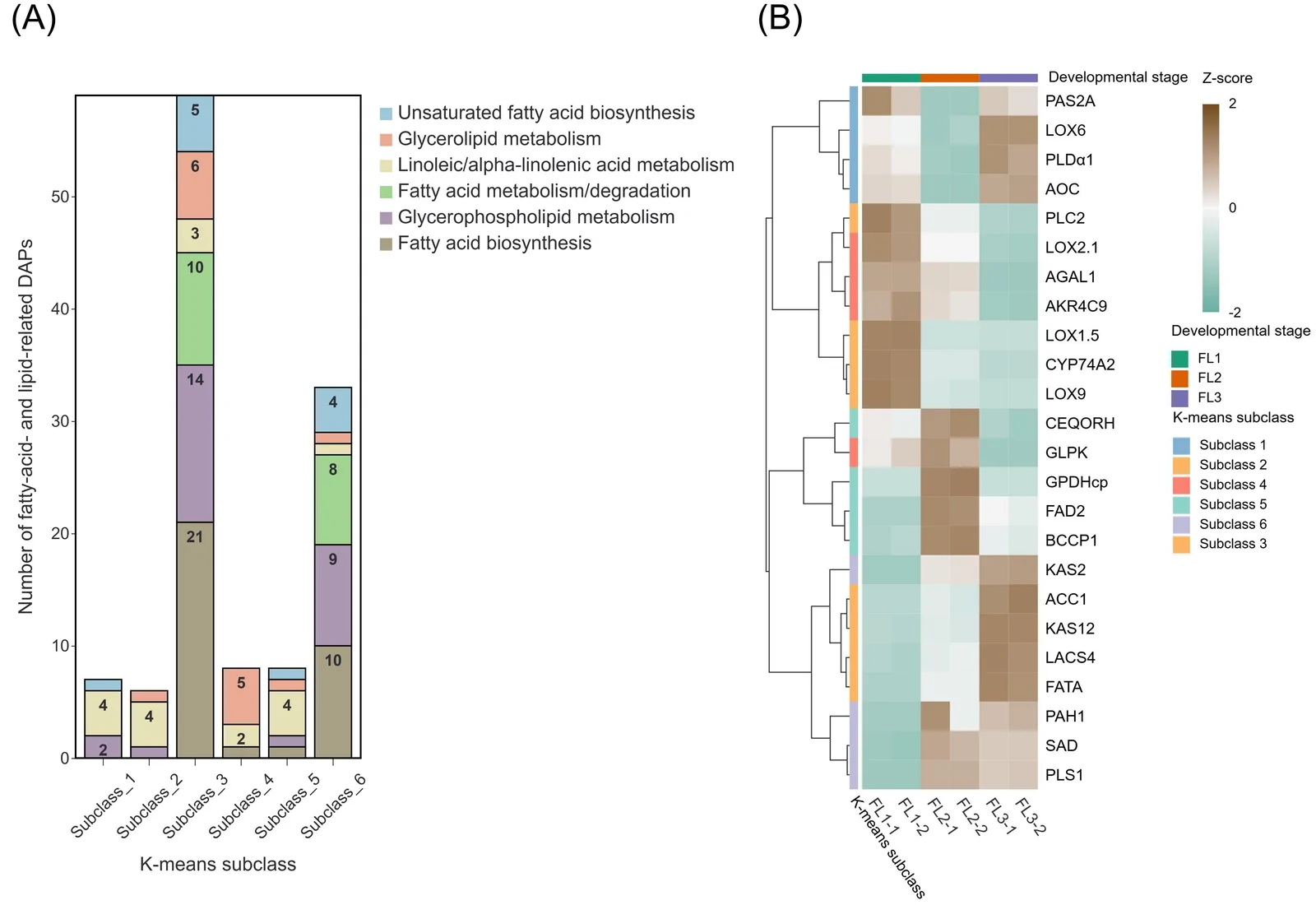
Developmental patterns of fatty-acid- and lipid-metabolism-related differentially abundant proteins in seedless oil palm mesocarp. **(A)** Distribution of fatty-acid- and lipid-metabolism-related DAPs among the six K-means subclasses. Stacked bars show the numbers of proteins assigned to different lipid-metabolism-related categories in each subclass. **(B)** Heatmap showing the developmental abundance patterns of selected fatty-acid- and lipid-metabolism-related proteins across FL1, FL2, and FL3. The annotation bar indicates the K-means subclass assigned to each protein. Proteomic analysis included two biological replicates per developmental stage.

Representative proteins showed distinct developmental abundance patterns (**Figure 9B**). KAS12, FATA, and LACS4 showed marked increases at FL3, whereas SAD and PLS1 exhibited late-stage increasing patterns. In contrast, FAD2-like showed relatively higher abundance at FL2.

### 3.9 K-means clustering of descriptive transcriptomic patterns during seedless oil palm mesocarp development

K-means clustering divided the 7,490 genes into six subclasses with distinct developmental FPKM patterns across FL1, FL2, and FL3 (**Figure 10**). Subclasses 1–6 contained 1,134, 1,771, 656, 562, 1,908, and 1,459 genes, respectively. Genes in Subclass 1 showed a progressive decrease from FL1 to FL3, whereas Subclasses 2 and 3 exhibited relatively higher transcript abundance at FL2. Subclass 4 showed lower transcript abundance at FL2 followed by recovery at FL3, while Subclass 5 exhibited a progressive increase and reached the highest level at FL3. Genes in Subclass 6 showed relatively higher transcript abundance at FL1 followed by decreased levels at FL2 and FL3.

**Figure 10:**
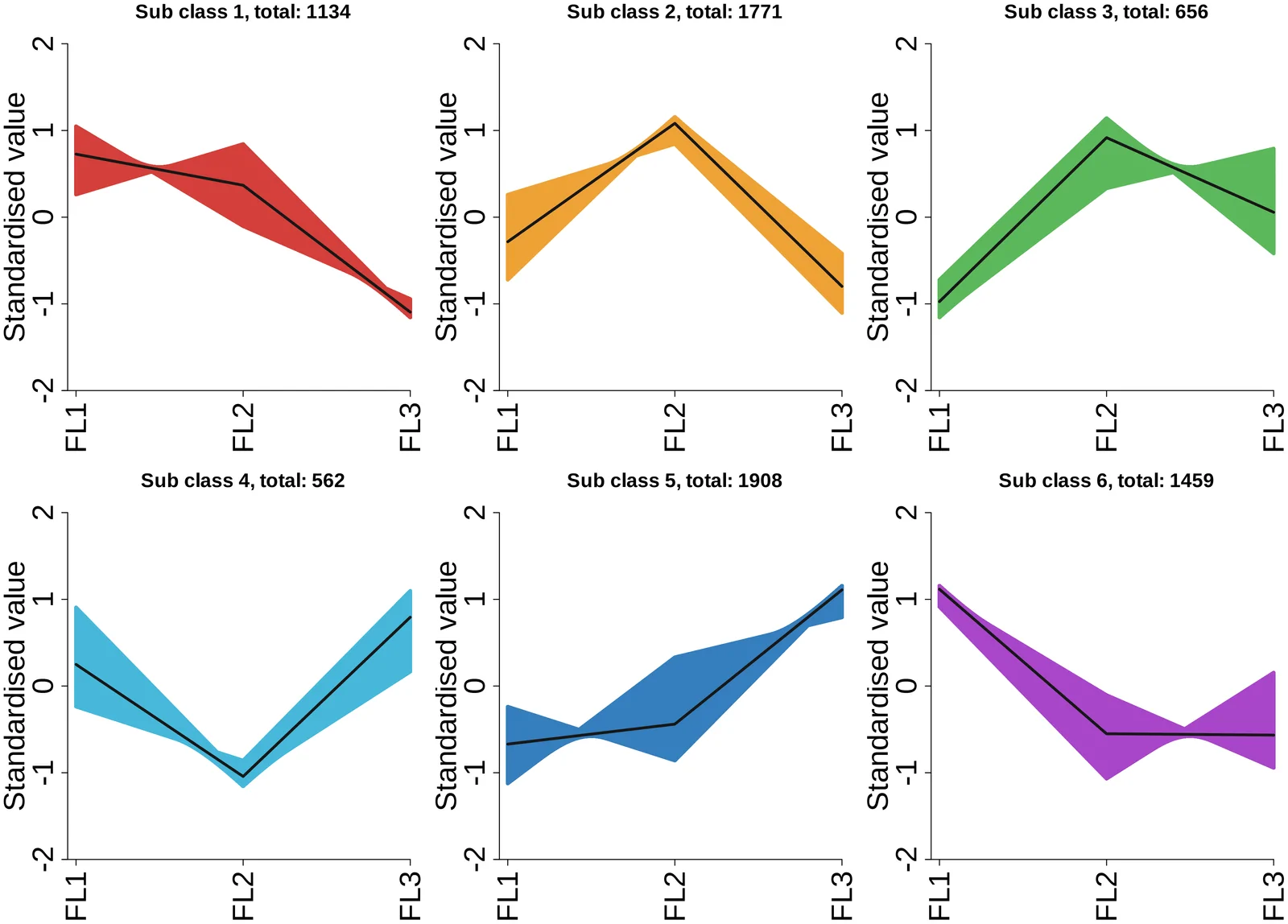
K-means clustering of 7,490 genes based on standardized FPKM values across FL1, FL2, and FL3. The number in each panel indicates the genes assigned to the corresponding subclass, and the vertical axis represents standardized FPKM values. RNA-seq was based on one biological sample per developmental stage.

### 3.10 Developmental patterns of fatty-acid- and lipid-metabolism-related genes in seedless oil palm

A total of 127 genes were annotated to fatty-acid- and lipid-metabolism-related KEGG pathways and were distributed across all six K-means subclasses (**Figure 11A**). Subclass 5 contained the largest number of these genes (56), followed by Subclass 2 (28), Subclasses 4 and 6 (13 each), Subclass 1 (12), and Subclass 3 (5). These genes were mainly associated with fatty acid biosynthesis, biosynthesis of unsaturated fatty acids, fatty acid metabolism/degradation, glycerolipid metabolism, glycerophospholipid metabolism, and linoleic/α-linolenic acid metabolism.

**Figure 11:**
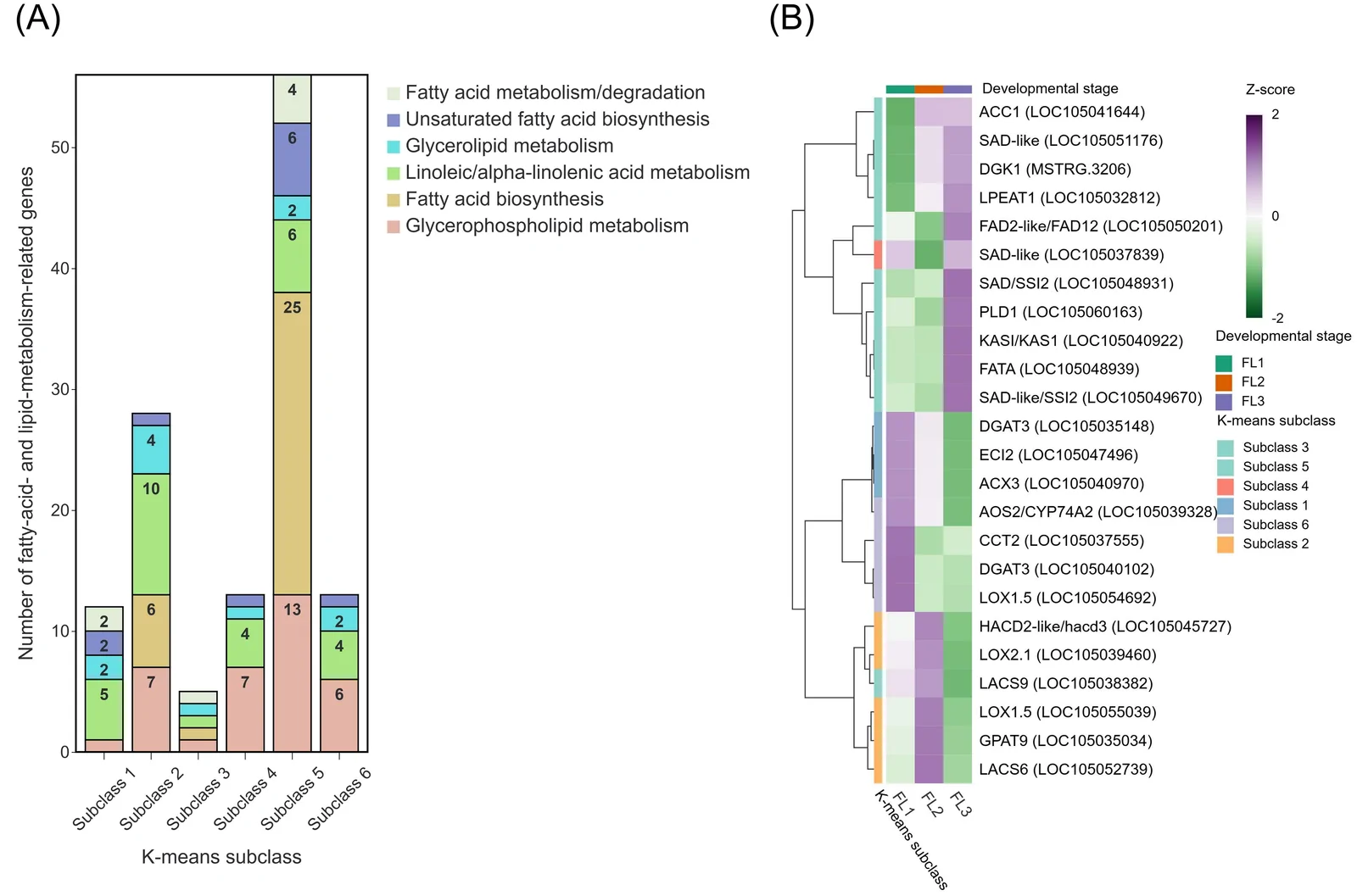
Developmental patterns of fatty-acid- and lipid-metabolism-related genes in seedless oil palm mesocarp. **(A)** Distribution of 127 lipid-metabolism-related genes among the six K-means subclasses. Stacked bars indicate the numbers of genes assigned to each metabolic category. **(B)** Heatmap showing the developmental FPKM patterns of selected fatty-acid- and lipid-metabolism-related genes across FL1, FL2, and FL3. The annotation bar indicates the K-means subclass of each gene. RNA-seq was based on one biological sample per developmental stage.

Selected fatty-acid- and lipid-metabolism-related genes showed distinct developmental FPKM patterns across FL1, FL2, and FL3 (**Figure 11B**). *KAS1/KASI*, *SAD/SSI2*, *FATA*, and *FAD2-like* were assigned to Subclass 5 and showed relatively higher FPKM values at FL3. Other genes shown in **Figure 11B** were distributed among the remaining subclasses and exhibited FL1-high, FL2-high, or recovery-type developmental patterns.

### 3.11 Integrated pathway-level comparison of metabolomic, transcriptomic, and proteomic datasets during seedless oil palm mesocarp development

KEGG pathway analysis was performed on the metabolomic and proteomic datasets (**Figure 12**). After multiple-testing correction, 14 proteomic pathways remained significantly enriched, including fatty acid biosynthesis, fatty acid metabolism, glycolysis/gluconeogenesis, pyruvate metabolism, starch and sucrose metabolism, and carbon metabolism. In contrast, no metabolomic pathways reached the FDR significance threshold.

**Figure 12:**
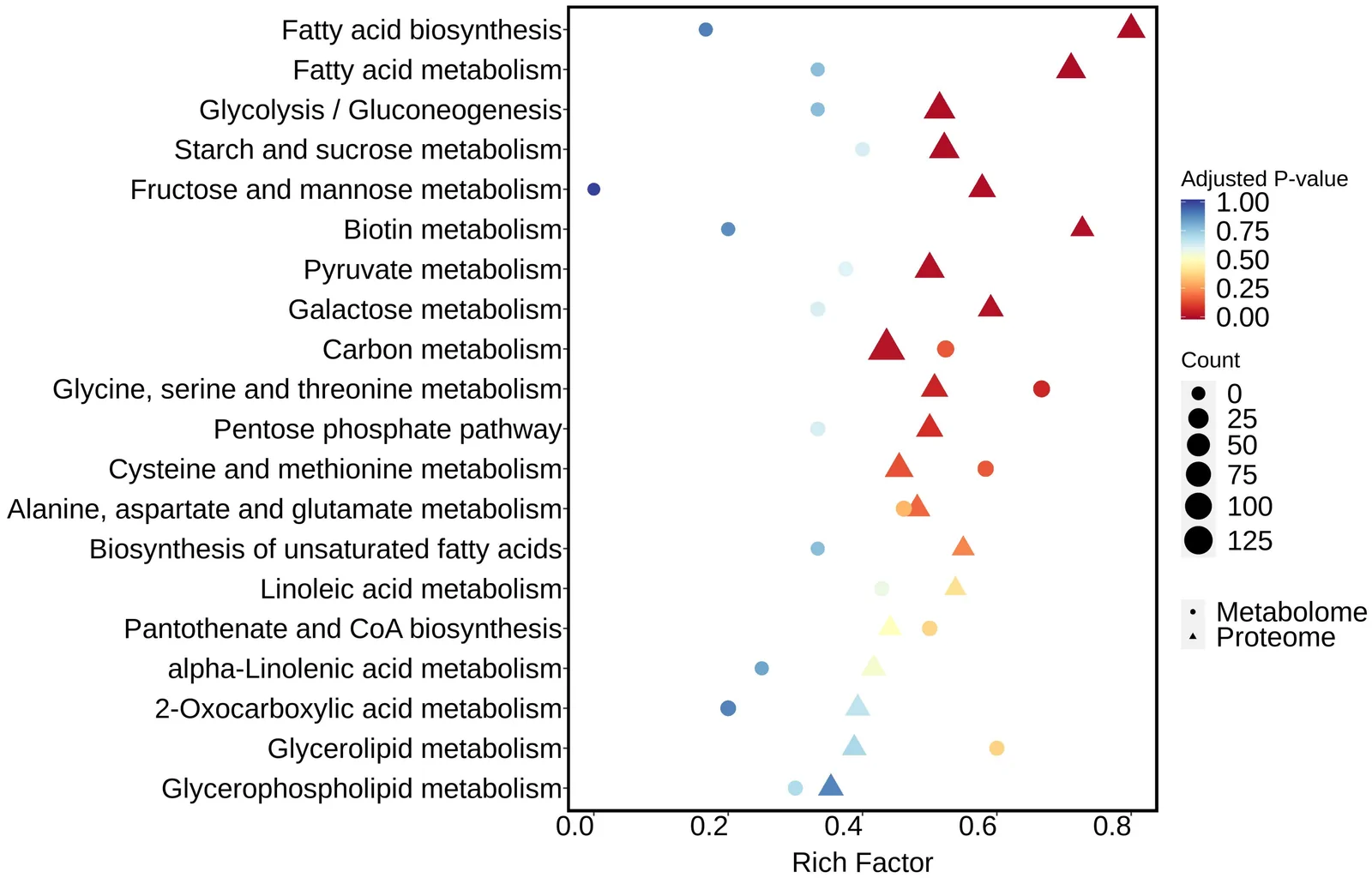
KEGG pathway enrichment analysis of metabolomic and proteomic datasets during seedless oil palm mesocarp development. FDR-screened differentially accumulated metabolites (DAMs) and differentially abundant proteins (DAPs) were subjected to KEGG pathway enrichment analysis. The horizontal axis represents the rich factor, symbol size indicates the number of pathway-associated metabolites or proteins, and symbol color represents the Benjamini–Hochberg-adjusted P value. Circles and triangles represent the metabolomic and proteomic datasets, respectively. Twenty representative pathways associated with central carbon and lipid metabolism are shown.

To compare pathway representation across the three omics layers, annotated metabolites, genes, and proteins were summarized for 20 representative metabolic pathways (**Figure 13**). Carbon metabolism, glycolysis/gluconeogenesis, pyruvate metabolism, fatty acid biosynthesis and metabolism, biosynthesis of unsaturated fatty acids, glycerolipid metabolism, and glycerophospholipid metabolism were represented across multiple omics datasets. For example, carbon metabolism contained 11 metabolites, 152 genes, and 129 proteins, whereas fatty acid biosynthesis contained 1 metabolite, 36 genes, and 44 proteins.

**Figure 13:**
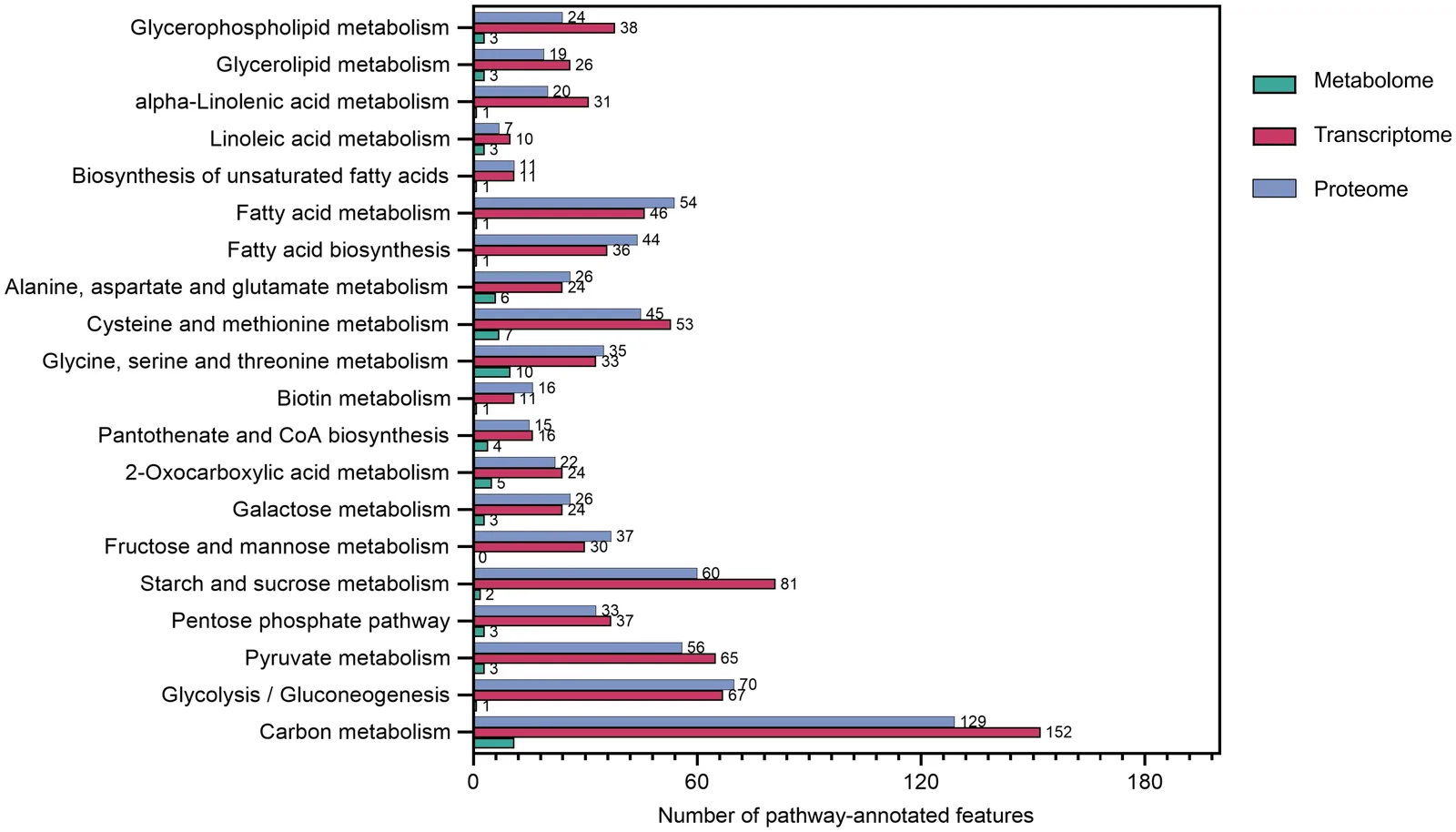
Pathway representation across metabolomic, transcriptomic, and proteomic datasets during seedless oil palm mesocarp development. The numbers of pathway-annotated differentially accumulated metabolites (DAMs), genes, and differentially abundant proteins (DAPs) are shown for 20 representative metabolic pathways. Transcriptomic counts were derived from the 7,490 genes retained for developmental FPKM analysis. The selected pathways are mainly associated with central carbon metabolism, fatty acid biosynthesis and metabolism, unsaturated fatty acid biosynthesis, glycerolipid metabolism, and glycerophospholipid metabolism.

### 3.12 Integrated pathway model of fatty acid and lipid metabolism associated with oleic acid accumulation

An integrated pathway model was constructed based on metabolomic, transcriptomic, and proteomic data to summarize fatty-acid- and lipid-metabolism-related genes, proteins, and metabolites associated with oleic acid accumulation during seedless oil palm mesocarp development (**Figure 14**). The model included precursor supply, plastidial fatty acid biosynthesis and desaturation, fatty acid release and activation, and downstream glycerolipid and glycerophospholipid metabolism.

**Figure 14:**
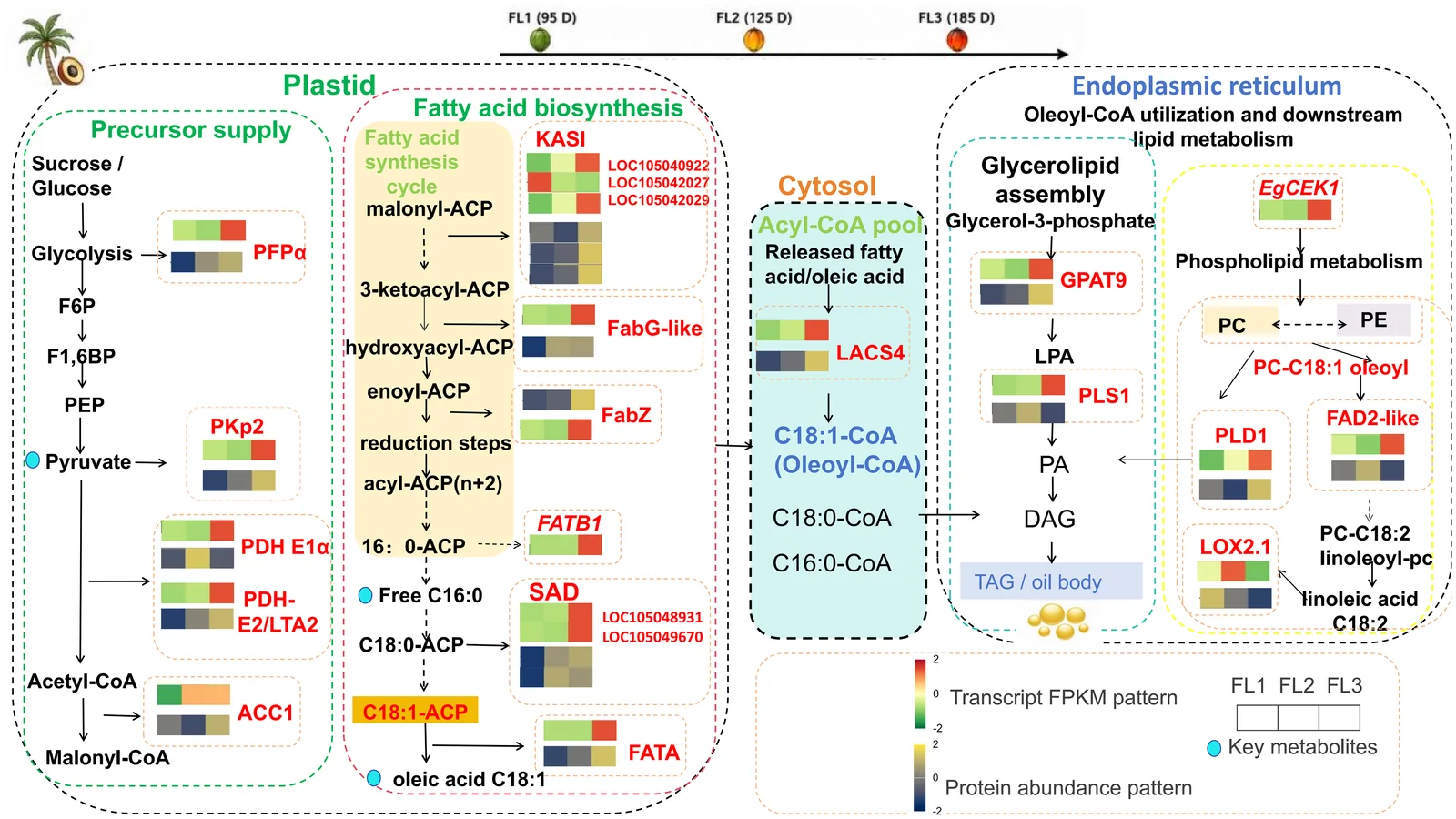
Integrated multi-omics pathway model associated with oleic acid accumulation in seedless oil palm mesocarp. Selected fatty-acid- and lipid-metabolism-related genes and proteins, together with key metabolites detected in the metabolomic dataset, were mapped to pathways involving precursor supply, plastidial fatty acid biosynthesis and desaturation, fatty acid release and activation, glycerolipid metabolism, and glycerophospholipid metabolism. Red labels indicate the selected pathway-related genes or proteins, and blue circles indicate detected metabolites, including pyruvate, palmitic acid (C16:0), oleic acid (C18:1), and linoleic acid (C18:2). Other metabolites and intermediates are shown to illustrate the pathway framework. For each displayed factor, the upper heatmap represents the transcript FPKM pattern and the lower heatmap represents the normalized protein abundance pattern across FL1 (95 days after pollination), FL2 (125 days after pollination), and FL3 (185 days after pollination), where corresponding data were available. Arrows indicate the proposed metabolic relationships among pathway components.

In the precursor-supply pathway, PFPα and PKp2 were associated with glycolytic carbon conversion, PDH E1α and PDH-E2/LTA2 with acetyl-CoA formation, and ACC1 with malonyl-CoA formation. In plastidial fatty acid biosynthesis, KASI, FabG-like, and FabZ were associated with fatty acid chain elongation and subsequent reduction and dehydration reactions, whereas SAD was positioned at the C18:0-ACP-to-C18:1-ACP desaturation step. FATB1 and FATA were associated with acyl-ACP hydrolysis and fatty acid release.

Following fatty acid release, LACS4 was associated with long-chain fatty acid activation. GPAT9 and PLS1 were associated with glycerolipid assembly, with PLS1 positioned at the conversion of lysophosphatidic acid (LPA) to phosphatidic acid (PA). PLD1 and *EgCEK2-like* were associated with glycerophospholipid metabolism, whereas FAD2-like and LOX2.1 were associated with downstream C18 unsaturated fatty acid and linoleic acid metabolism. The integrated transcript and protein profiles showed distinct developmental patterns among these pathway-related factors, including a late-stage increase in SAD protein abundance and a relatively higher FAD2-like protein abundance at FL2, highlighting developmental differences between C18:1 formation and its downstream desaturation.

PFPα, pyrophosphate-dependent phosphofructokinase alpha subunit; PKp2, plastidic pyruvate kinase2; PDH E1α, pyruvate dehydrogenase E1 alpha subunit; PDH-E2/LTA2, dihydrolipoyllysine-residue acetyltransferase component of the pyruvate dehydrogenase complex; ACC1, acetyl-CoA carboxylase 1; KASI, beta-ketoacyl-ACP synthase I; FabG-like, 3-oxoacyl-ACP reductase-like protein; FabZ, 3-hydroxyacyl-ACP dehydratase; FATB1, acyl-ACP thioesterase B1; SAD, stearoyl-ACP desaturase; FATA, oleoyl-ACP thioesterase; LACS4, long-chain acyl-CoA synthetase 4; GPAT9, glycerol-3-phosphate acyltransferase 9; PLS1, 1-acyl-sn-glycerol-3-phosphate acyltransferase (lysophosphatidic acid acyltransferase, LPAAT); PLD1, phospholipase D1; EgCEK2-like, probable choline kinase 2; FAD2-like, endoplasmic-reticulum omega-6 fatty acid desaturase 2-like; LOX2.1, lipoxygenase 2.1.

### 3.13 qRT-PCR analysis of candidate gene expression patterns

Nine candidate genes associated with precursor supply, fatty acid biosynthesis, desaturation, acyl release, and lipid metabolism were analyzed by qRT-PCR across FL1, FL2, and FL3 (**Figure 15**). *SAD* showed significantly higher expression at FL3 than at FL1 and FL2, whereas *FATB1* showed significantly higher expression at FL2 and FL3 than at FL1. *FabZ* also showed significantly higher expression at FL2 and FL3 than at FL1. No significant differences among developmental stages were observed for *PKp2*, *PFPα*, *PDH E1α*, *KAS12*, *ACC1*, or *EgCEK2-like*.

**Figure 15:**
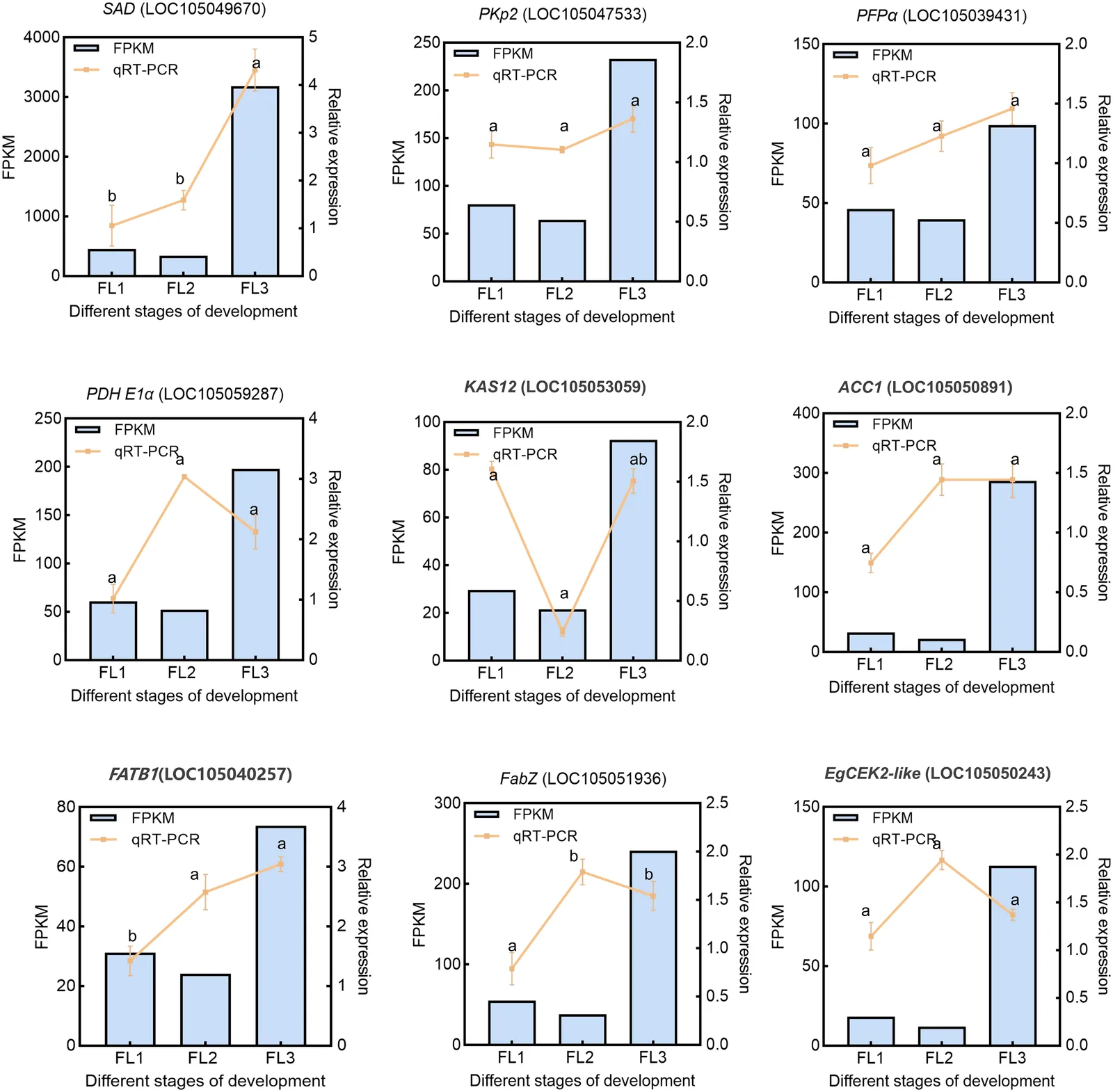
Comparison of transcriptomic FPKM and qRT-PCR expression patterns of selected candidate genes during seedless oil palm mesocarp development. Blue bars represent transcriptomic FPKM values, and orange lines represent relative expression levels determined by qRT-PCR. qRT-PCR data are presented as mean ± SD of three biological replicates per developmental stage. Different lowercase letters indicate significant differences in qRT-PCR expression among developmental stages according to one-way ANOVA followed by Tukey’s multiple-comparison test (P < 0.05). RNA-seq was based on one biological sample per developmental stage, and the FPKM values are therefore presented descriptively.

Comparison with the corresponding transcriptomic FPKM patterns showed broadly similar developmental trends for some genes, particularly *SAD* and *FATB1*, which exhibited higher expression at the late developmental stage in both datasets. Other genes showed partially different developmental patterns between the transcriptomic and qRT-PCR results. Among the nine genes analyzed, *SAD* showed the most pronounced increase in qRT-PCR expression at FL3.

## 4 Discussion

### 4.1 Metabolic characteristics associated with oleic acid accumulation in seedless oil palm

The normalized LC–MS/MS signal corresponding to oleic acid increased markedly at FL3, showing a pronounced late-stage increase during seedless oil palm mesocarp development. These measurements represent relative LC–MS/MS signal changes rather than absolute fatty-acid concentrations or conventional fatty-acid composition. This developmental pattern is consistent with previous studies showing that oleic acid is a major unsaturated fatty acid in oil palm mesocarp and undergoes substantial changes during fruit development (Xu et al., 2025a, 2025b).

At the proteomic level, significantly enriched pathways included fatty acid biosynthesis, fatty acid metabolism, glycolysis/gluconeogenesis, pyruvate metabolism, starch and sucrose metabolism, and carbon metabolism. The metabolomic dataset also contained metabolites mapped to several carbon- and lipid-metabolism-related pathways, although these pathways did not remain significantly enriched after multiple-testing correction. Together, these results suggest that the late-stage increase in the oleic-acid-associated signal is accompanied by broader changes in fatty acid biosynthesis, precursor metabolism, and lipid metabolism.

### 4.2 Genes and proteins associated with precursor supply for fatty acid biosynthesis

Plastidial fatty acid biosynthesis requires acetyl-CoA, malonyl-CoA, reducing power, and energy. Therefore, glycolysis, pyruvate metabolism, and central carbon metabolism provide important upstream support for fatty acid synthesis. Previous multi-omics studies in oil-accumulating tissues have also revealed close associations between carbon metabolism and lipid accumulation during development (Liu et al., 2024). In the present study, these metabolic processes were represented across the metabolomic, proteomic, and transcriptomic datasets.

Among the candidate genes, PFPα is involved in the reversible conversion between fructose-6-phosphate and fructose-1,6-bisphosphate, whereas PKp2 homologs catalyze the conversion of phosphoenolpyruvate to pyruvate, linking glycolysis with pyruvate supply (Andre et al., 2007; Duan et al., 2016). PDH E1α and DLAT are components of the pyruvate dehydrogenase complex involved in pyruvate-to-acetyl-CoA conversion (Tovar-Mendez et al., 2003), while ACC1 participates in acetyl-CoA carboxylation and malonyl-CoA formation, a key step in fatty acid biosynthesis (Sasaki and Nagano, 2004). Together, these components are associated with upstream carbon metabolism and acetyl-CoA/malonyl-CoA supply, suggesting a potential link between precursor metabolism and the late-stage increase in the oleic-acid-associated signal.

### 4.3 Fatty acid biosynthetic cycle and genes/proteins associated with oleic acid formation

*De novo* fatty acid biosynthesis in plants occurs primarily in plastids and involves a series of enzymes responsible for malonyl-CoA utilization, fatty acid chain elongation, reduction, dehydration, desaturation, and acyl release. KAS, FabG, FabZ, and related enzymes participate in successive reactions of the fatty acid biosynthetic cycle (Brown et al., 2009; Li-Beisson et al., 2013; Bu et al., 2023; Afifi et al., 2025).

In the present study, KAS1/KASI, FabG-like, and FabZ were identified as candidate proteins associated with chain elongation, reduction, and dehydration during fatty acid biosynthesis.

Among the genes and proteins associated with oleic acid formation, SAD is particularly relevant because stearoyl-ACP desaturase catalyzes the conversion of C18:0-ACP to C18:1-ACP, a key step in plant oleic acid biosynthesis (Hernandez et al., 2019; Zhu et al., 2024). In the present study, SAD showed significantly higher qRT-PCR expression at FL3 than at FL1 and FL2, consistent with the late-stage increase in the oleic-acid-associated LC–MS/MS signal. This developmental pattern further highlights the association of SAD with oleic acid formation during seedless oil palm mesocarp development.

FATB1 is functionally distinct from SAD. Whereas SAD is involved in C18:0-ACP-to-C18:1-ACP desaturation, FATB-type acyl-ACP thioesterases participate in acyl release from ACP and may influence fatty acid chain composition and acyl availability (Bonaventure et al., 2003). Together, these genes and proteins represent successive biochemical steps involved in fatty acid synthesis, oleic acid formation, and acyl release.

### 4.4 Genes and proteins associated with acyl activation and downstream lipid metabolism

Following oleic acid formation and acyl release, free C18:1 can be activated to oleoyl-CoA and subsequently enter glycerolipid and glycerophospholipid metabolism. Previous studies have shown that storage lipid synthesis and membrane lipid metabolism are closely associated with acyl activation, glycerolipid assembly, and lipid remodeling, involving enzymes such as LACS, GPAT, DGAT, PDAT, and PLD (Regmi et al., 2020; Bhandari and Bates, 2021; Li et al., 2024; Parchuri et al., 2024; Cai et al., 2025). Dynamic lipid remodeling has also been observed in seedless oil palm fruits, further indicating active changes in lipid metabolic processes in this material (Liu et al., 2025).

In this study, LACS4 was associated with long-chain fatty acid activation, whereas GPAT9 and PLS1 were positioned at sequential steps of glycerolipid assembly. GPAT9 catalyzes the initial acylation of glycerol-3-phosphate to form lysophosphatidic acid (LPA), whereas PLS1 (LOC105033594), annotated as a 1-acyl-sn-glycerol-3-phosphate acyltransferase (lysophosphatidic acid acyltransferase, LPAAT), was associated with the subsequent conversion of LPA to phosphatidic acid (PA). PA represents an important intermediate in glycerolipid metabolism and also participates in multiple physiological processes in plants (Yao and Xue, 2018). PLD1 was associated with phospholipid metabolism and remodeling. EgCEK2-like (LOC105050243), annotated as a probable choline kinase 2, was associated with choline phosphorylation and phosphatidylcholine-related metabolism. The choline/ethanolamine kinase family has also been characterized in Arabidopsis, supporting the functional annotation of this candidate (Rabeler et al., 2022).

Together, these components are associated with fatty acid activation, glycerolipid assembly, and phospholipid metabolism and may participate in the downstream utilization of C18:1-derived acyl groups during seedless oil palm mesocarp development.

### 4.5 C18-unsaturated fatty acid metabolism associated with oleic acid accumulation

Oleic acid represents both a product of plastidial fatty acid desaturation and a substrate for further C18-unsaturated fatty acid metabolism. SAD catalyzes the conversion of C18:0-ACP to C18:1-ACP, whereas FAD2 catalyzes the further desaturation of phosphatidylcholine-bound C18:1 to C18:2. Previous studies have shown that developmental regulation of genes involved in unsaturated fatty acid biosynthesis is closely associated with changes in C18-unsaturated fatty acid accumulation in oil-producing plants (Meng et al., 2021; Shi et al., 2021). Therefore, oleic acid accumulation may be influenced by both C18:1 formation and its subsequent desaturation and utilization.

In the present study, SAD and FAD2-like showed distinct developmental patterns. At the protein level, SAD exhibited a late-stage increase, whereas FAD2-like showed relatively higher abundance at FL2. Their corresponding transcriptomic FPKM profiles also showed different developmental patterns. Linoleic acid displayed a developmental LC–MS/MS signal pattern distinct from that of oleic acid. In addition, LOX2.1 was associated with downstream linoleic-acid metabolism, through which C18:2 may enter oxylipin-related or fatty-acid-turnover processes (Yu et al., 2019; Sandalio et al., 2019; Savchenko et al., 2022; Sugimoto et al., 2022; Koley et al., 2025).

These results suggest that late-stage oleic acid accumulation may be associated with the balance between SAD-mediated C18:1 formation and subsequent FAD2-mediated desaturation and downstream utilization. The relative contributions of these processes remain to be further determined.

### 4.6 Multi-omics integration for prioritizing candidate genes and proteins associated with oleic acid accumulation

Multi-omics integration linked candidate genes and proteins to successive metabolic processes associated with precursor supply, fatty acid biosynthesis, C18:0-ACP-to-C18:1-ACP desaturation, acyl release and activation, and downstream glycerolipid/glycerophospholipid metabolism. Among these candidates, SAD was associated with the C18:0-ACP-to-C18:1-ACP desaturation step and showed a late-stage increase in transcriptomic, proteomic, and qRT-PCR profiles. KAS-related candidates, FabG-like, FabZ, PDH E1α, DLAT, ACC1, LACS4, PLS1, and PLD1 were associated with other steps of fatty acid biosynthesis, precursor supply, acyl activation, and downstream lipid metabolism. FAD2-like and LOX2.1 further extended the integrated pathway to C18-unsaturated fatty acid and linoleic acid metabolism. Importantly, among the nine genes examined by qRT-PCR, only *SAD, FATB1*, and *FabZ* showed significant developmental differences, whereas *PKp*2, *PFPα*, *PDH E1α*, *KAS12*, *ACC1*, and *EgCEK2-like* did not. These six candidates should therefore be regarded as being supported by pathway position and descriptive multi-omics patterns rather than by statistically significant qRT-PCR changes.

Previous oil palm transcriptomic and metabolomic studies identified *SAD, FabD, LACS6, BC, FabB*, and *FabI* as candidate genes associated with oleic acid accumulation (Xu et al., 2025a, 2025b). *SAD* was also identified in the present study, while additional similarities were observed at the pathway and enzyme-family levels. For example, *LACS6* and *LACS4* are both associated with long-chain fatty acid activation, BC- and ACC-related components participate in acetyl-CoA carboxylation, and FabB/FabI together with KAS-related candidates, FabG-like, and FabZ are involved in different steps of plastidial fatty acid biosynthesis. These similarities indicate partial convergence between the present results and previous studies, while differences in specific candidate genes may reflect differences in genetic background, developmental stage, and analytical design.

An important extension of the present study is the incorporation of proteomic information into the analysis of oleic acid accumulation. Fourteen proteomic KEGG pathways remained significantly enriched after multiple-testing correction, including pathways related to fatty acid biosynthesis, fatty acid metabolism, glycolysis/gluconeogenesis, pyruvate metabolism, and carbon metabolism. The protein abundance patterns therefore provided an additional layer of information for interpreting developmental changes associated with precursor supply, fatty acid synthesis, acyl activation, and downstream lipid metabolism. Integrated omics approaches have also been widely applied to characterize lipid accumulation in other oil-producing species. Transcriptomic and metabolomic analyses have identified developmental changes in lipid biosynthesis in soybean and walnut (Xu et al., 2022; Liang et al., 2023), while a recent multi-omics analysis of Camellia oleifera revealed coordinated changes in pathways associated with triacylglycerol and fatty acid accumulation (Wang et al., 2025).

Similar developmental regulation of fatty acid metabolism has also been reported in olive mesocarp (Tsitsekian et al., 2025) identified transcriptional modules associated with changes in saturated, monounsaturated, and polyunsaturated fatty acids and highlighted potential roles of SAD4, PDAT1, hormonal signaling, and transcription factors during fruit ripening. These findings provide a broader context for further investigation of metabolic and regulatory processes associated with oleic acid accumulation in oil palm.

### 4.7 Limitations

Several limitations of this study should be considered. RNA-seq was performed using one biological sample per developmental stage; therefore, the transcriptomic data represent descriptive developmental FPKM patterns rather than statistically supported differential expression. Metabolomic and proteomic analyses were based on two biological replicates per developmental stage. Although Benjamini–Hochberg multiple-testing correction was applied, the limited number of biological replicates reduced the statistical power of these analyses. Although the proteomic biological replicates showed high within-stage abundance correlations and relatively low median CVs, the use of only two biological replicates limits the estimation of biological variance. Therefore, the large number of DAPs, particularly in comparisons involving FL3, should be interpreted cautiously and confirmed in future studies with larger biological replication.

In addition, the fatty-acid-related data were obtained using untargeted HILIC LC–MS/MS and represent normalized relative signals rather than absolute fatty acid concentrations or conventional fatty acid composition. Further validation using GC–FID, GC–MS, or targeted lipidomic approaches will therefore be necessary. The amplification efficiencies of the qRT-PCR primers were not independently determined using serial-dilution standard curves. Moreover, although the reference genome assembly was identified as E. guineensis GCF_000442705.1, the exact gene-annotation release and KEGG database version used in the original analysis could not be reliably recovered.

The developmental design of this study primarily captures temporal changes within seedless oil palm mesocarp. In the absence of a contrasting genotype analyzed at matched developmental stages, it remains difficult to distinguish oleic-acid-associated changes from broader ripening-related processes. Accordingly, the candidate pathways, genes, and proteins identified here should be interpreted mainly as developmental features associated with oleic acid accumulation. Comparative analyses with tenera or other reference genotypes at corresponding developmental stages would help further resolve genotype-associated regulatory features from common mesocarp maturation responses. In addition, the present multi-omics analysis does not directly determine metabolic flux or regulatory relationships. Further studies incorporating replicated transcriptomics, targeted fatty acid quantification, comparative genotype analysis, and functional characterization of prioritized candidates will be needed to clarify their specific roles in oleic acid accumulation.

Finally, the multi-omics analysis identified candidate pathways, genes, and proteins associated with oleic acid accumulation but did not directly determine metabolic flux or regulatory relationships. Further studies using replicated transcriptomics, targeted fatty acid quantification, and functional characterization of prioritized genes and proteins will be required to clarify their roles in oleic acid accumulation.

## 5 Conclusions and perspectives

This study integrated metabolomic, proteomic, transcriptomic, and qRT-PCR analyses across three developmental stages of seedless oil palm mesocarp to investigate metabolic pathways and candidate genes and proteins associated with oleic acid accumulation. The oleic-acid-associated LC–MS/MS signal increased markedly at FL3. Multi-omics analysis highlighted fatty acid biosynthesis, fatty acid metabolism, unsaturated fatty acid biosynthesis, glycolysis/gluconeogenesis, pyruvate metabolism, carbon metabolism, glycerolipid metabolism, and glycerophospholipid metabolism as major pathways associated with this developmental process.

Genes and proteins were identified at successive metabolic steps from precursor supply and fatty acid biosynthesis to acyl activation and downstream lipid metabolism. Among these candidates, SAD was associated with the C18:0-ACP-to-C18:1-ACP desaturation step, and its transcriptomic and qRT-PCR expression patterns showed a pronounced late-stage increase, consistent with the increase in the oleic-acid-associated signal. KAS-related candidates, FabG-like, FabZ, PDH E1α, DLAT, ACC1, LACS4, FATA, GPAT9, PLS1, PLD1, and EgCEK2-like were associated with fatty acid biosynthesis, precursor supply, acyl activation, and glycerolipid/glycerophospholipid metabolism, whereas FAD2-like and LOX2.1 were associated with downstream C18-unsaturated fatty acid metabolism.

Among the nine genes examined by qRT-PCR, *SAD, FATB1*, and *FabZ* showed significant developmental differences, whereas *PKp2*, *PFPα*, *PDH E1α*, *KAS12, ACC1*, and *EgCEK2-like* did not. The latter candidates therefore remain supported mainly by pathway annotation and descriptive transcriptomic or proteomic patterns, while other candidates were prioritized from the multi-omics analysis but were not examined by qRT-PCR.

## Data Availability

The metabolomics dataset generated in this study has been deposited in MetaboLights under accession number MTBLS15725. The transcriptomic sequencing data have been deposited in the NCBI Sequence Read Archive (SRA) under BioProject accession number PRJNA1529219. The mass spectrometry proteomics data have been deposited to the ProteomeXchange Consortium via the PRIDE partner repository with dataset identifier PXD084281.
